# Understanding and Neutralizing the Expense Prediction Bias: The Role of Accessibility, Typicality, and Skewness

**DOI:** 10.1177/00222437211068025

**Published:** 2022-02-15

**Authors:** Ray Charles “Chuck” Howard, David J. Hardisty, Abigail B. Sussman, Marcel F. Lukas

**Keywords:** expense prediction bias, consumer financial decision making, budgeting, accessibility, typicality, skewness

## Abstract

Consumers display an expense prediction bias in which they underpredict their future spending. The authors propose this bias occurs in large part because (1) consumers base their predictions on typical expenses that come to mind easily during prediction, (2) taken together, typical expenses lead to a prediction near the mode of a consumer's expense distribution rather than the mean, and (3) expenses display positive skew (with mode < mean). Accordingly, the authors also propose that prompting consumers to consider reasons why their expenses might be different than usual increases predictions—and therefore prediction accuracy—by bringing atypical expenses to mind. Ten studies (N = 6,044) provide support for this account of the bias and the “atypical intervention” developed to neutralize it.

Consumers regularly predict their future expenses (Peetz et al. 2016), and the accuracy of these predictions can be highly consequential (Berman et al. 2016). In particular, underpredicting future expenses can be costly. For example, the most common reason consumers withdraw money early from their 401(k) retirement savings accounts is to cover unpredicted expenses, and these breaches cost consumers approximately $7 billion a year in penalties (Fellowes and Willemin 2013). Similarly, almost 2 million Americans per year use a payday loan to cover unpredicted expenses (Pew Charitable Trusts 2012), and the annual interest rate on these loans frequently exceeds 400% (Consumer Federation of America 2018). Many consumers also expect that they will be able to pay off their credit card balance each month (Yang, Markoczy, and Qi 2007), yet American consumers collectively hold over $1 trillion in credit card debt and pay the associated interest costs (Federal Reserve Bank of New York 2018).

These examples suggest that increasing expense prediction accuracy can help consumers spend, save, and/or borrow money more efficiently. For example, if consumers had a clearer idea of how much money they will spend in the future, it could encourage them to spend less in the present to avoid the costs associated with borrowing to cover expenses down the road. The prosocial value of helping consumers avoid these costs is self-evident. The rush by venture capitalists to fund personal finance apps that help consumers manage their expenses (CB Insights 2018) indicates that there is also firm value in improving expense prediction accuracy.

Echoing these real-world examples, academic research also suggests that consumers tend to underpredict their future expenses (Peetz and Buehler 2009; Sussman and Alter 2012; Ülkümen, Thomas, and Morwitz 2008), a phenomenon we label the “expense prediction bias.” The goal of the present research is to understand how the cognitive (in)accessibility of certain expenses contributes to this bias, then leverage that insight to develop and test a practical intervention that improves consumers’ expense prediction accuracy. We theorize that consumers’ expense predictions are based on *typical* expenses, because these expenses come to mind most easily during prediction. Taken together, typical expenses lead to a prediction that is closer to the mode of a consumer's expense distribution than to the mean.^1^ This results in underprediction because, generally speaking, consumers’ expense distributions are skewed to the right, with mode < mean. Accordingly, we hypothesize that prompting consumers to consider reasons why their upcoming expenses might be *different* than usual will increase predictions—and therefore prediction accuracy—by bringing atypical expenses to mind.

**Table 1.** Results of the Think-Aloud Protocol Pilot Study.

Classification	Proportion	First Thought	Examples
Typical	83.64%	67.27%	“**Typically** I buy groceries every week. That's about $50 dollars or so.” “On **average**, I would say I spend around $10 per day on food and drinks.” “**Normally** I will spend, uh, approximately $20 per day for food.” “On Friday I **usually** get gas so that's usually thirty dollars a week.”
Future-oriented	54.55%	32.73%	“Huh, I’m traveling **next week** too, traveling is…I’ll say $400, yeah.” “**This Sunday**, I might go to the mall to get new work clothes for my co-op, so that might be dress shoes, that might be maybe $120.” “Are there any birthdays **coming up**? Oh wait, my brother's birthday…that's going to be about $300.”
Adjustment	50.91%	0.00%	“I'll put about $20 for like **miscellaneous** items.” “And just for **miscellaneous** items I would put another $10.” “And then, shopping…**miscellaneous**, we’ll just budget $50 for that.”

We next outline the expense prediction process. We then develop our theory and hypotheses and present ten studies that test them. We conclude by discussing the implications of our work for theory and practice.

## The Expense Prediction Process

Expense prediction requires answering some variation of the question “How much will I spend in the next week (or next month)?”^2^ We posit that consumers answer this question in one of two ways: they either use a “bottom-up” approach to prediction in which they begin by generating a list of specific expenses^3^ and estimate how much they will spend on each (e.g., “Groceries are usually $150, gas for the car is typically $60, and Netflix is $15, so my total expenses for next week will be $225”), or they use a “top-down” approach in which they call to mind a holistic dollar amount that attempts to capture total spending (e.g., “I usually spend around $225 each week, therefore my expenses will total $225 next week.”). To determine which of these approaches is more common, we conducted a preregistered survey (https://aspredicted.org/mq26m.pdf) that asked 184 U.S. consumers on Prolific Academic (M_age_ = 30.80 years; 56.5% female) to predict their total spending for the next week, then indicate whether they had taken a bottom-up approach (“I started by thinking of individual expenses and adding them together”), a top-down approach (“I started by thinking of a number for my total spending”), or an alternative approach (“Other—Please Describe”). We found that 60.30% of participants reported using a bottom-up approach (different than 50%: z = 2.79, *p* = .005, 95% confidence interval [CI_95%_] = [52.84%, 67.42%]), 37.50% reported using a top-down approach (different than 50%: z = 3.39, *p* = .001, CI_95%_ = [30.49%, 44.92%]), and 2.20% of participants indicated “Other.” We therefore focus on the bottom-up approach in our theorizing below, but Web Appendix A explains how our theoretical framework can be applied to the top-down prediction process as well.

## The Cognitive Accessibility of Typical Expenses

Consumers’ expenses fall along a continuum from typical to atypical (Heath and Soll 1996). We conceptualize typical expenses as more regularly occurring expenditures like a weekly trip to the grocery store or putting gas in the car. Typical expenses tend to happen at relatively standard intervals (e.g., daily, weekly, monthly), and the amount of these expenses is relatively stable across intervals (e.g., if your weekly grocery bill is typically $150, you might spend $125 in some weeks and $175 in others, but it is unlikely you will spend $0 or $1,000). In contrast, atypical expenses are irregular expenditures such as a medical bill or home repair. Atypical expenses tend to happen at relatively abnormal intervals (e.g., twice in one week then not again for several months), and the amount of these expenses can vary substantially (e.g., copay for a prescription might be $10, whereas the cost of a major surgery can be exponentially larger). Thus, atypical expenses are unusual in occurrence, amount, or both. This conceptualization is consistent with research showing that perceived typicality is a function of both exposure frequency and representativeness (e.g., Boush and Loken 1991; Sussman, Paley, and Alter 2021).

In the present research, we propose that expense predictions are largely based on typical past expenses, because it is far easier to think of typical past expenses than it is to predict actual future expenses. To illustrate this point, consider that a comprehensive expense prediction requires anticipating all possible future expenses, estimating the amount of each expense and the probability it will occur, then adding these probability-weighted amounts together. In contrast, typical expenses are easily learned, categorized, and remembered (Heath and Soll 1996). More generally, when a person is repeatedly exposed to variations of a stimulus—as is the case with typical, recurring expenses—mental representations of what is typical can be encoded with relative ease, and they remain highly accessible in memory thereafter (Kahneman 2003; Rosch and Mervis 1975). This makes our proposition that expense predictions are based on typical past expenses consistent with the finding that cognitive accessibility often determines the content of judgments and decisions (Johnson, Häubl, and Keinan 2007; Tversky and Kahneman 1974).

If our theorizing is correct and consumers base their predictions on typical expenses, then a bottom-up approach to determining “How much will I spend next week?” essentially involves answering two closely related questions: (1) "What do I typically buy?" and (2) "How much do those things typically cost?" This possibility is consistent with research showing that typical exemplars (e.g., individual expenses) and prototypes (e.g., generalized amounts) influence judgments in similar ways, and that they often operate in unison (Ross and Makin 1999; Verbeemen et al. 2007). It is also consistent with the observation that when people are confronted with challenging questions, they often unconsciously substitute the answer to easier questions instead (Kahneman 2011). Thus, when people need to answer the difficult question, “How much will I spend?,” they instead answer the easier question, “How much do I *typically* spend?”

To explore our proposition that predictions are based on typical expenses we ran a think-aloud protocol study with 55 undergraduate students from a large Canadian university. Each participant was taken to a private room where they received written instructions to say aloud every thought that came to mind as they predicted their expenses for the next week. Participants’ thoughts were recorded and later transcribed and coded. Specifically, research assistants independently coded transcriptions for references to typical expenses, future oriented expenses (i.e., expenses specific to the next week), and adjustments for unexpected expenses. The research assistants also coded which of these thoughts appeared first in each transcript.

Table 1 presents the proportion of participants who referenced each type of expense, the proportion who mentioned each type of expense first, and verbatim examples. A significantly higher proportion of participants referenced typical expenses than the proportion of participants who referenced future-oriented expenses (χ^2^(1) = 10.80, *p* = .001, CI_95%_ = [11.88%, 44.12%]). Typical expenses also came to mind first for a strong majority of participants (z = 2.56, CI_95%_ = [53.29%, 79.32%], *p* = .010). Finally, only half of participants made an adjustment for unexpected expenses. Taken together, these results support our proposition that predictions are based on typical expenses that come to mind easily during prediction. Notably, these results were replicated in the control condition of Supplemental Study A (see Web Appendix F), which was preregistered and conducted with a nonstudent sample for greater generalizability.

## Why “Typical” Expense Predictions Are Biased: The Role of Skew

**Figure 1.**
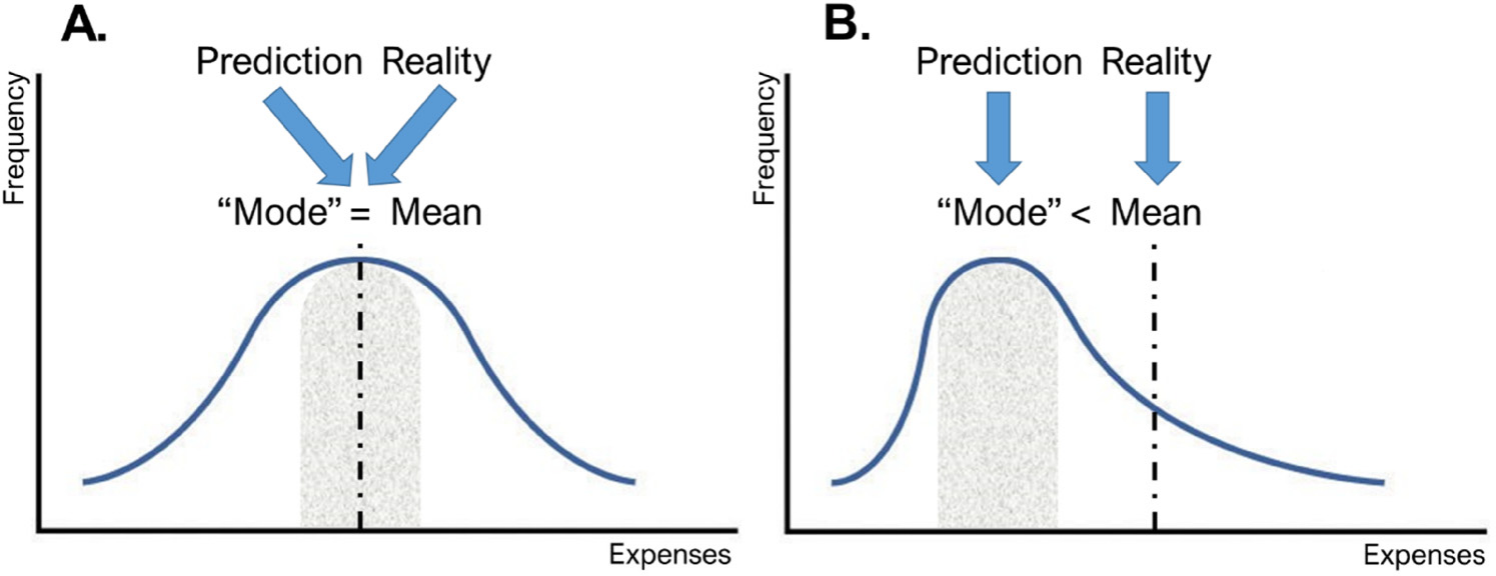
Predictions around the “mode” of a distribution lead to accurate predictions relative to the mean in a normal distribution but lead to systematic underprediction relative to the mean in a positively skewed distribution.

*Notes:* The mode is represented by the gray-shaded region; the mean is represented by the dashed line.

Normally, it is advisable to use relevant past experience to predict future outcomes (Buehler, Griffin and Peetz 2010). However, in the case of expense prediction, consumers who base their predictions on typical past expenses will be systematically biased toward underpredicting their future expenses. Why does this happen? The shape of the distribution of expenses is a critical factor. When consumers base their predictions on typical, frequently occurring expenses at typical amounts, this translates into predictions near the mode of their expense distribution (the most frequent outcome). If expenses are normally distributed with mode = mean, then predictions based on typical expenses will be relatively accurate (Figure 1, Panel A). However, if expenses are positively skewed with mode < mean, then predictions based on typical expenses will result in underprediction (Figure 1, Panel B).

In the present research, we propose that predictions based on typical expenses cause consumers to underpredict their future spending because the distribution of consumer expenses is generally skewed to the right with mode < mean. The expectation that expenses are skewed to the right can be derived logically from the observation that expenses are bounded by zero on the left but free to run as high as a consumer's credit will allow on the right. Importantly, it is also supported empirically by the longitudinal field data we collected for Studies 1 and 5. For example, the distribution of total weekly expenses for the average consumer in Study 1 displays skewness of 1.03, which meets the standard definition of a “highly skewed” variable (Bulmer 1979). Next, we explain how skew leads to bias at three interrelated levels of prediction: (1) which individual expenses will occur?, (2) how much will each expense cost?, and (3) what will the total weekly amount be?

1. *Which individual expenses will occur?* Suppose Kavita wants to predict which individual expenses she will need to pay for next week. Like all consumers, Kavita has a distribution of possible expenses that may or may not occur: $60 for gas, $200 for groceries, $550 for a new refrigerator, and so on. If Kavita's possible individual expenses are normally distributed (with moderately sized expenses occurring most frequently and atypically high and low expenses evenly distributed on either side), and if Kavita is more likely to predict the occurrence of typical than atypical expenses, her total expense prediction will still be accurate, because her typical expenses together comprise a modal amount that is near the mean (Figure 1, Panel A). In contrast, if Kavita's possible expenses are positively skewed, such that atypical expenses are generally higher expenses, Kavita will underpredict her mean total expenses because her typical expenses together comprise a mode that is below the mean (Figure 1, Panel B).
2. *How much will each expense cost?* Once Kavita has predicted that an individual expense will occur, she needs to predict how much it will cost. For example, suppose Kavita predicts she will buy gas next week. In a typical (modal) week gas costs her $60, so she predicts $60 for next week. If her weekly spending on gas is normally distributed, with the amount sometimes higher than $60 but equally often lower than $60, then Kavita's prediction will match the mean amount she spends on gas and be accurate (Figure 1, Panel A). However, if her weekly spending on gas is positively skewed with the amount more likely to be higher than $60 than less, Kavita will underpredict her mean spending because mode < mean (Figure 1, Panel B).
3. *What will the total weekly amount be?* Finally, imagine a distribution of total weekly expense amounts: some weeks Kavita spends $400 in total, some weeks she spends $600 in total, but most frequently she spends $500 in total. If Kavita predicts typical individual expenses will occur ( # 1) at typical amounts ( # 2), these add up to a typical or modal prediction of total weekly expenses. For example, Kavita might know that in a typical week she spends $60 on gas, $40 on drinks, and $400 on food, and therefore, she would add these up and predict $500 in total, which would be her typical weekly expense amount and would be the mode of her weekly expense distribution. Following the same logic as mentioned previously, if the distribution of weekly expenses is normally distributed, Kavita's weekly prediction based on typical expenses will be accurate (Figure 1, Panel A). However, if the distribution of weekly expenses is positively skewed, then Kavita will underpredict because a correct prediction of mean weekly expenses must include some atypical expenses (Figure 1, Panel B).

As illustrated in these three cases, a prediction based on typical expenses can lead to bias when there is distributional skew at any level of expense prediction. Of course, the three levels are interrelated: weekly expense totals ( # 3) are made up of the occurrence of individual expenses ( # 1), each of which may have a smaller or larger amount ( # 2). We next introduce the hypotheses we derived from this account of the expense prediction bias.

## Hypotheses

Under the general case of positively skewed expenses with mode < mean, our theorizing leads to the expectation that consumers display an expense prediction bias in which they underpredict their future expenses. It also produces two novel hypotheses regarding the relationship between expenses and their perceived typicality. One implication of our theory is that expense predictions do not account for atypical expenses. In contrast, retrospection is more deeply grounded in reality (Kane, Van Boven, and Peter McGraw 2012), which implies that expense recall will include both typical and atypical expenses. Therefore, if our theorizing is correct, we should find support for the hypothesis that consumers display a typicality bias in which they predict their future expenses will be more typical than their past expenses (H_1a_).

Our theorizing also implies that the more heavily a consumer relies on typical expenses when making their prediction, the lower their expense prediction will be. This follows from the general case that expenses are positively skewed: if typical expenses together constitute the mode of total spending, then a more typical prediction will generally be a lower prediction, because the mode of a positively skewed distribution is usually lower than most other points on the distribution. In contrast, if typical expenses coincide with the mean of the distribution, then a more typical prediction would generally be a higher prediction, because the mean of a positively skewed distribution is usually higher than most other points on the distribution. It is therefore informative to test the hypothesis that perceived typicality of future expenses is negatively correlated with expense predictions (H_1b_).

If the expense prediction bias occurs in part because consumers make predictions based on typical expenses, then it follows that helping consumers bring *atypical* expenses to mind during prediction will increase their prediction accuracy. We propose that having people consider reasons why their expenses might be different than usual will serve as a simple intervention that accomplishes this goal. Accordingly, we hypothesize that prompting consumers to consider reasons why their future expenses might be different from a typical week (or month) increases expense predictions and therefore increases expense prediction accuracy (H_2_).

Our intervention is designed to improve prediction accuracy under the general case of positively skewed expenses. However, from a theoretical perspective, it is useful to consider what would happen if the distribution of expenses were manipulated to be normally distributed rather than positively skewed. All else equal, our theorizing suggests that a normal distribution of expense amounts (e.g., with mean = median = mode = $200) should produce higher predictions than a positively skewed distribution (e.g., with mean and median = $200, but mode = $190). We therefore used a lab paradigm to manipulate skew and test the hypothesis that expense predictions are higher and closer to the mean when the distribution of expense amounts is normally distributed versus positively skewed, all else equal (H_3a_). Likewise, in a preregistered field study, we test the hypothesis that expense prediction is more accurate in an expense category (groceries) with lower skew than in a category (online shopping) with higher skew (H_3b_). Thus, our theory predicts *when* consumers will show more or less bias in their predictions.

Our theorizing also implies that skewness should moderate the effectiveness of the atypical intervention versus control. When expenses are positively skewed, thinking of reasons why they will be different than usual means thinking of individual expenses that make total weekly spending higher than usual. But, when expenses are more normally distributed, “different than usual” means “higher than usual” (e.g., an anniversary dinner on date night with your spouse) or “lower than usual” (e.g., missing date night with your spouse because you get sick) with equal probability. We therefore hypothesize that the atypical intervention increases predictions (vs. control) in expense categories with relatively strong positive skew (e.g., online shopping) more than in categories with relatively moderate positive skew (e.g., grocery shopping) (H_4_).

## Overview of Studies

Study 1 examines the magnitude, persistence, and prevalence of the expense prediction bias and tests H_1_–H_2_ in a repeated-measures longitudinal field study. Study 1 also examines the association between expense prediction bias and several theoretically relevant individual differences, including prediction confidence, savings goals, and trait optimism. Study 2 replicates and extends Study 1 by testing H_1_–H_2_ in a nationally representative sample of U.S. consumers and demonstrating that the atypical intervention increases expense predictions by increasing the cognitive accessibility of atypical expenses. Study 3 examines the possibility that our intervention operates by increasing the diagnosticity of atypical expenses in addition to (or rather than) their accessibility (Tybout et al. 2005). Taken together, the results of Studies 2 and 3 indicate that the atypical intervention affects predictions by increasing the accessibility of atypical expenses more than their diagnosticity. Study 4 tests H_3a_ by experimentally manipulating skewness and showing that, all else equal, predictions are lower when expenses are positively skewed with mode < mean than when normally distributed with mode = mean. Study 5 tests H_3b_ and H_4_ in a field experiment conducted with users of a popular personal finance app, and it identifies skewness as an informative boundary condition of the intervention. Furthermore, in a set of four supplemental studies we also (1) conceptually replicate Study 2 and directly replicate the think-aloud pilot study, (2) conceptually replicate Study 4, (3) show that our theoretical framework applies to monthly as well as weekly expense predictions, and (4) establish that the atypical intervention increases intentions to save as well as predictions.

## Study 1: A Longitudinal Field Study of Expense Prediction Bias

The first goal of Study 1 was to test H_1_–H_2_ in a repeated-measures longitudinal field study. The second goal was to examine the relationship between expense predictions and individual differences such as prediction confidence (Ülkümen, Thomas, and Morwitz 2008), motivation to save (Peetz and Buehler 2009), and trait optimism. The third goal was to contribute a more comprehensive understanding of the expense prediction bias itself, by observing its magnitude, persistence, and prevalence within a highly engaged nonstudent sample across multiple points in time. To accomplish these goals, we partnered with Vancity, Canada's largest community credit union, to run a five-week longitudinal field study with a sample of its members.

### Method

**Figure 2.**
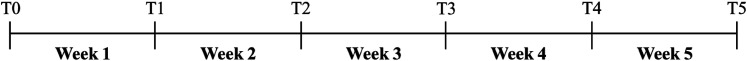
Data collection schedule in Study 1.

Participants in this field study were members of Vancity, a Canadian credit union with approximately 500,000 members. Participants were recruited through an online panel of roughly 5,000 members that Vancity uses to conduct research. We targeted a sample size of 200 based on effect sizes observed in pilot studies. Each participant completed six surveys over the course of five weeks, as illustrated by each time period marked in Figure 2.

Because we had no prior experience sampling from this population, data collection took place in two waves (for details and rationale, see Web Appendix B). At the end of both waves of data collection, we had complete data from 187 participants (M_age_ = 51.12 years; 57.8% female). Compensation for each participant included a personalized spending report (provided at the end of the study) that served as an incentive to predict and report expenses as accurately as possible. Participants also received a $10 Amazon gift certificate for each completed survey.

All surveys were emailed to participants at noon on a Sunday and required completion before 11:59 p.m. the next day. The first survey asked participants to predict their expenses for the next week as follows: “Please take some time to estimate your total expenses for the next week. By ‘total expenses’ we mean everything you will pay for during the next week. [Page Break] Please enter your estimated total expenses for the next week.” We then measured perceived typicality of predicted expenses by asking participants, “How different or similar do you think your expenses will be for the next week, relative to a typical week?” (1 = “very different,” and 7 = “very similar”). We also measured prediction confidence by asking, “How sure or confident are you that your estimate of your total expenses for the next week is accurate?” (1 = “very unsure,” and 7 = “very sure”), so that we could test the hypothesis that prediction confidence and expense predictions are negatively correlated (Ülkümen, Thomas, and Morwitz 2008). Finally, participants answered the same prediction, typicality, and confidence questions with respect to the next month.

The remaining five surveys began by asking participants to log into their online bank account and report their expenses for the past week, then predict their expenses for the next week. Both expense reports and predictions were followed by the same measures of perceived typicality and confidence used in survey 1. In the second-to-last survey (i.e., at T4), half of the sample was randomly assigned to receive the atypical intervention, making the final week of the study a 2 (condition: control vs. atypical) × 2 (expenses: predicted vs. reported) between-within design.^4^ In the atypical intervention condition, participants received the following instructions before making their prediction: “Please take some time to consider why your expenses for the next week might be different from a typical week. In the spaces provided below, please type 3 reasons why your expenses for next week might be different from a typical week.” Participants in the control condition received the same prediction instructions as in the previous surveys.

Across the six surveys we also measured the following individual differences: savings goals (Peetz and Buehler 2009), trait optimism (Scheier, Carver, and Bridges 1994), short-term financial propensity to plan (Lynch et al. 2010), numeracy (Schwartz et al. 1997), spendthrift–tightwad tendencies (Rick, Cryder, and Loewenstein 2008), openness to experience (John, Donahue, and Kentle 1991), temporal discounting (Kirby and Maraković 1996), and cyclical versus linear time orientation (Tam and Dholakia 2014). In addition, we collected exploratory measures related to behaviors such as budgeting, borrowing, and spending. None of these measures were found to be consistently or significantly correlated with the expense prediction bias, and these null results are presented in Web Appendix B.

As highlighted in our theoretical development, the distribution of expenses displays significant positive skew. This is a defining characteristic of the data, and an important element in our theory; it also presents challenges for responsible inferential analysis in Studies 1 and 2, where we compare predicted expenses to reported expenses across experimental conditions. To address this, we exclude the data of outlier participants whose reported expenses exceed their predictions by a factor of 10 or more (or vice versa), then natural log–transform the distributions of reported and predicted expenses. For ease of interpretation, we then exponentiate our descriptive results and present them in dollar terms. Notably, this procedure ameliorates concerns related to homogeneity of variance assumption violations and the influence of outliers, but it does not change the pattern of results observed in the raw data. To illustrate this, we present a pair of robustness tests (Winsorization and nonparametric median analysis) in Web Appendix C, and we detail the impact that the transformation process has on the expense data in Web Appendix D.

### Results

#### Weekly expense prediction bias

**Figure 3.**
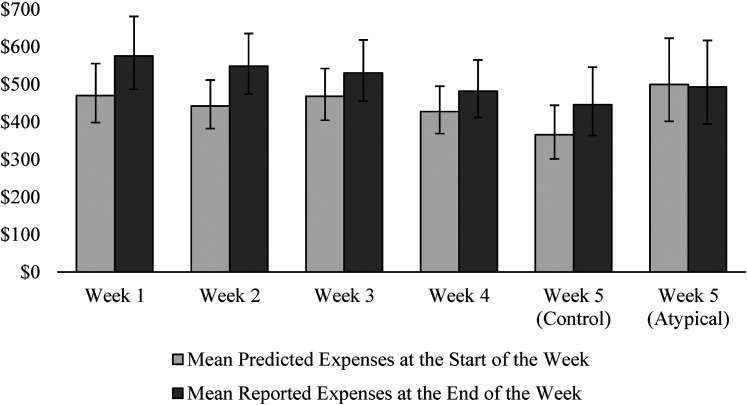
Weekly expense prediction accuracy in Study 1.

*Notes:* Error bars represent 95% CIs.

We calculated weekly expense prediction bias as the difference between predicted expenses at the start of each week and reported expenses at the end of each week. As illustrated in Figure 3 and Table 2, predicted expenses were significantly lower than reported expenses in each week of the study, except in the atypical condition during week 5, when our intervention neutralized the expense prediction bias. A 2 (condition: control vs. atypical) × 2 (expenses: predicted vs. reported) between-within analysis of variance (ANOVA) confirmed a significant condition × expenses interaction (F(1, 181) = 5.08, *p* = .025, $\eta_{p}^{2}$ = .027). Planned contrasts further confirmed that predicted expenses were 36.7% higher in the atypical condition than in the control condition (F(1, 181) = 4.48, *p* = .036) and that reported expenses did not differ significantly between the two conditions (F(1, 181) = .44, *p* = .51). In dollar terms, expense prediction bias in the control condition was −$79.99 (different from zero, t(91) =  −3.19, *p* = .002), but only $6.65 in the atypical condition (not different from zero, t(90) = .20, *p* = .85).

**Table 2.** T-Test Results for Prediction Accuracy in Study 1.

	Week 1	Week 2	Week 3	Week 4	Week 5 (Control)	Week 5 (Atypical)
Mean bias	−$105.42	−$106.30	−$62.35	−$54.69	−$79.99	$6.65
t	−3.71	−4.35	−2.86	−2.73	−3.19	.19
d.f.	179	179	180	182	91	90
*p*	< .001	< .001	.005	.007	.002	.85

We next examined the extent to which predictions changed from week to week by performing a 2 (condition: control vs. atypical) × 5(week: 1 vs. 2 vs. 3 vs. 4 vs. 5) mixed-model ANOVA with condition as a between-subjects variable, week as a within-subject variable, and expense prediction as the dependent variable. There was no main effect of condition (F(1, 181) = .03, *p* = .86) or week (F(4, 178) = .88, *p* = .48), but there was a significant condition × week interaction (F(4, 178) = 6.16, *p* < .001, $\eta_{p}^{2}$ = .12). Contrast analysis further revealed that predictions only differed significantly between the two conditions when we introduced the intervention in week 5 and that predictions did not differ significantly from week 1 to week 4 (t(182) = 1.31, *p* = .19). This suggests that the tendency to base predictions on typical expenses is persistent. The same analysis with reported expenses as the dependent variable revealed no main effect of condition (F(1, 180) = .003, *p* = .95), a marginal main effect of week (F(4, 177) = 2.39, *p* = .052, $\eta_{p}^{2}$ = .05), and no condition × week interaction (F(4, 177) = 1.53, *p* = .20). Contrast analysis showed that reported expenses did not differ significantly between the two conditions in any week of the study (*p*s ≥ .30) but that spending in week 5 of the study was generally lower than in week 1 (t(182) = 2.34, *p* = .020). One potential explanation for this is that tracking expenses for the study may have made participants more aware of their spending and therefore more inclined to reduce it.

To examine the prevalence of underprediction, we compared the proportion of participants who underpredicted their expenses in each week of the study with the proportion who overpredicted. If predictions are based on typical expenses that comprise the mode, then in any given week we should observe that a higher proportion of participants underpredict than overpredict. This is because most points on a positively skewed distribution are usually higher than the mode, so a typical or modal prediction makes the probability of underpredicting higher than the probability of overpredicting. Consistent with this expectation, a significantly higher proportion of participants underpredicted than overpredicted in weeks 1–4 of the study, with the prevalence of underprediction ranging from 59.5% to 69.3% (*p*s ≤ .019). In week 5, the proportion of participants in the control condition who underpredicted was 66.2%, but the proportion of participants in the intervention condition who underpredicted was only 47.6% (χ^2^ (1) = 5.56, *p* = .018).

#### Monthly expense prediction bias

We calculated monthly expense prediction bias as the difference between participants’ monthly expense predictions at T0 and their reported expenses over the first four weeks of the study. Predicted expenses for the month (M_pred_ = $2,276.74, CI_95%_ = [2,031.64, 2,551.15]) were $416.77 or 15.5% lower than actual expenses incurred (M_actual_ = $2,693.51, 95% CI_95%_ = [2,376.06, 3,053.37]; t(184) = −3.85, *p* < .001). Furthermore, 63.2% of participants underpredicted their monthly expenses (z = 3.59, *p* < .001). To the best of our knowledge, this provides the first evidence that consumers underpredict their monthly expenses as well as their weekly expenses.

#### Perceived typicality

**Figure 4.**
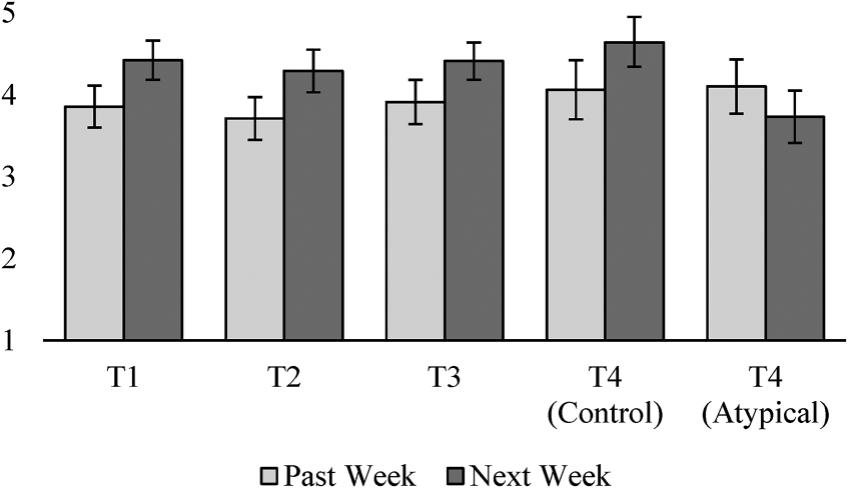
Mean typicality of past versus predicted expenses in each week (Study 1).

To test our hypothesis that consumers predict their future expenses will be more typical than their past expenses (H_1a_), we compared reported and predicted expense typicality at T1, T2, T3, and T4. In other words, we tested whether participants predicted their expenses would be more typical in week 2 than week 1, week 3 than week 2, and so on. As illustrated in Figure 4 and Table 3, participants predicted that their expenses would be more typical in the next (vs. past) week at all four points in time, until the atypical intervention neutralized this tendency at T4. Taken together, these results provide strong support for H_1a_.

**Table 3.** T-Tests Results Comparing Past Versus Predicted Typicality in Each Week of Study 1.

	T1	T2	T3	T4 (Control)	T4 (Atypical)
t	3.45	4.10	3.25	2.88	−1.78
d.f.	174	173	177	89	88
*p*	.001	< .001	.001	.005	.079
d	.26	.31	.24	.30	−.19

To test H_1b_, we analyzed the correlation between perceived typicality of future expenses and expense predictions. Perceived typicality of future expenses was negatively correlated with weekly expense predictions at T0 (r(185) = −.30, *p* < .001), T2 (r(185) = −.28, *p* < .001), and T4 (r(185) = −.25, *p* < .001). The correlation at T1 was marginally significant (r(185) = −.12, *p* = .09), as was the correlation between perceived typicality of monthly expenses and expense predictions for the month (r(185) = −.12, *p* = .09). The correlation at T3 was directionally consistent, though not significant (r(185) = −.03, *p* = .74). We next regressed predictions onto perceived typicality of future expenses and participant income to rule out the possibility that the correlation between predictions and typicality was due to wealthier participants with higher expenses also having more atypical expenses. This analysis produced only one meaningful change in these results: the relationship between perceived typicality and predictions at T1 became significant (*p* = .013). In aggregate, these findings support H_1b_.

#### Prediction confidence

We measured prediction confidence in this study because previous research suggests that lower prediction confidence leads people to adjust their expense predictions upward (Ülkümen, Thomas, and Morwitz 2008). If so, it should be the case that prediction confidence and expense prediction amount are negatively correlated in weeks 1–4 of the study. However, we found that the relationship between expense predictions and prediction confidence in weeks 1–4 was neither substantive (rs ≤ .073) nor significant (*p*s ≥ .31). Likewise, prediction confidence did not mediate the effect of experimental condition on predictions in week 5 of the study (indirect effect = .01, SE = (.03), CI_95%_ = [−.05, .06]).

### Discussion

The results of Study 1 offer longitudinal evidence from the field in support of H_1_–H_2_. Participants underpredicted their weekly and monthly expenses over the first four weeks of the study, but the atypical intervention neutralized this bias in week five (H_2_). Participants also consistently predicted that their future expenses would be more typical than their past expenses (H_1a_), and perceived typicality of future expenses was inversely related to predictions (H_1b_).

Study 1 also produced several meaningful null results (for details, see Web Appendix B). First, prediction confidence was not associated with bias, suggesting that these patterns are not explained by a meta-cognitive account of underprediction in which higher confidence leads to lower predictions (Ülkümen, Thomas, and Morwitz 2008). Study 1 also showed that the atypical intervention does not increase prediction accuracy by decreasing prediction confidence. Second, the presence of a savings goal was not associated with the bias, which indicates that underprediction in this study was not driven by motivated cognition in which the desire to save leads to unrealistically low predictions (Peetz and Buehler 2009). Third, trait optimism was not associated with the bias, which suggests that underprediction is not merely a function of an optimistic disposition.

## Study 2: The Accessibility of Atypical Expenses

The primary goal of Study 2 was to better understand the process by which our intervention improves prediction accuracy. Specifically, we examined the extent to which the intervention makes atypical expenses more cognitively accessible (vs. control), and the relationship between accessibility and predictions. We also used Study 2 to better understand the expense prediction process by including an experimental control condition that prompted participants to consider three reasons why their expenses would be similar to a typical week. Our expectation was that predictions and perceived typicality would not differ between the experimental control condition and the pure control condition, because if our theorizing is correct, then predictions in the pure control condition will already be based on typical expenses.

### Method

A nationally representative sample of 1,091 U.S. residents completed Study 2 via Time-Sharing Experiments for the Social Sciences (https://www.tessexperiments.org/). The recalled expenses of 43 participants exceeded their predictions by a factor of 10 or more (or vice versa), leaving us with an effective sample size of 1,048 (M_age_ = 49.59 years; 53.0% female; 72.8% Caucasian, 9.4% Black, 10.7% Hispanic, 7.2% Other; Mode level of education = Bachelor's degree; Median household annual income = $50,000–$59,999).

Participants were randomly assigned to one of three conditions: pure control, experimental control (i.e., the “typical condition”), or intervention (i.e., the “atypical condition”). Participants in the pure control condition and atypical intervention condition predicted and recalled their weekly expenses for the next and past week as in Study 1. Participants in the typical condition received the same prediction instructions as participants in the atypical condition, but rather than list three reasons why their expenses might be different from a typical week, we asked them to list three reasons why their expenses might similar. We hypothesized that expense predictions and perceived typicality would not differ significantly between the typical condition and the control condition, because if our theorizing is correct, then predictions in the control condition should already be based on typical expenses. The order of prediction and recall was counterbalanced in each condition.^5^

We next presented participants with an atypical expense-listing task that asked, “Is there anything you believe you will spend money on in the next week that you did NOT spend money on during the past week?” and “Is there anything that you spent money on during the past week that you believe you will NOT spend money on in the next week?” Participants were then given the opportunity to list a description and corresponding dollar amount for up to five such expenses. Our principal expectation was that our intervention would make it easier to retrieve atypical expenses for the next week, which would result in a higher number of expenses being listed in the atypical condition as compared with the control and typical conditions. Furthermore, we expected that the number of atypical expenses listed for the next week would mediate the relationship between experimental condition and predicted expenses.

Finally, participants completed the same measures of perceived typicality used in Study 1, and five exploratory measures designed to explore the relationship between predictions, financial slack (Zauberman and Lynch 2005), various measures of spending (e.g., willingness to pay for an optional expense such as a fancy dinner out with friends), and available resources. These exploratory measures yielded null results that are discussed in Web Appendix E. We applied the same data exclusion and transformation criteria in Study 2 that we used in Study 1. Robustness test results are available in Web Appendices C and D.

### Results

#### Replicating Study 1

The results of Study 1 were replicated in the control condition of Study 2. Participants predicted their future expenses would be 9.00% lower than their past expenses (M_nextweek_ = $215.47, CI_95%_ = [$195.00, $238.06]; M_pastweek_ = $236.77, CI_95%_ = [$214.52, $261.36]; t(415) = −2.76, *p* = .006), participants predicted that their future expenses would be more typical than their past expenses (M_nextweek_ = 4.65, CI_95%_ = [4.48, 4.81]; M_pastweek_ = 4.40, CI_95%_ = [4.23, 4.57]; t(415) = 3.42, *p* < .001, d = .17), higher perceived typicality of future expenses was associated with lower expense predictions (r(414) = −.21, *p* < .001), and this association remained significant after controlling for participant income (B = −.14, SE = .03, t(414) = −4.97, *p* < .001). We next expand our analyses to examine expenses and perceived typicality across all three conditions.

#### Predicted versus recalled expenses

Predicted expenses were 9.00% ($21.31) lower than recalled expenses in the control condition (t(415) = −2.76, *p* = .006) and 6.39% ($13.63) lower than recalled expenses in the typical condition (t(331) = −2.07, *p* = .039). However, predicted expenses did not differ significantly from recalled expenses in the atypical condition (t(299) = 1.49, *p* = .14). In other words, the tendency to predict that expenses would be lower in the future than the past was neutralized by the atypical intervention (H_2_). A 3 (condition: control vs. typical vs. atypical) × 2 (time period: past week vs. next week) between-within ANOVA with expenses as the dependent variable confirmed a significant main effect of condition (F(2, 1,045) = 4.61, *p* = .010), no main effect of time period (F(1, 1,045) = 1.67, *p* = .20), and a significant condition × time period interaction (F(2, 1,045) = 5.21, *p* = .006). In support of H_2_, planned contrasts confirmed that predicted expenses in the atypical condition (M_atypical_ = $271.16, CI_95%_ = [$239.15, $307.42]) were 25.85% ($55.69) higher than in the control condition (M_control_ = $215.47, CI_95%_ = [$195.00, 238.06]; t(1,045) = 2.90, *p* = .004), and 35.72% ($71.36) higher than in the typical condition (M_typical_ = $199.80, CI_95%_ = [$179.18, $222.78]; t(1,045) = 3.66, *p* < .001). Predictions did not differ between the control and typical conditions (t(1,045) = .98, *p* = .33), which is consistent with our proposition that consumers naturally make typical expense predictions. Planned contrasts also revealed that recalled expenses did not differ significantly between the atypical (M_atypical_ = $251.84, CI_95%_ = [$222.49, $285.09]) and control (M_control_ = $236.77, CI_95%_ = [$214.52, $261.36]; t(1,045) = .78, *p* = .43) conditions, but they were somewhat lower in the typical condition (M_typical_ = $213.43, CI_95%_ = [$191.41, $237.98]) than in the atypical condition (t(1,045) = 2.00, *p* = .046).^6^ Notably, this makes our test of the difference between predicted and recalled expenses in these two conditions conservative, because lower (higher) recalled expenses decreases (increases) the difference. However, despite lower recalled expenses in the typical condition and higher recalled expenses in the atypical condition, we observe a significant difference in the former but not in the latter.

#### Atypical expense-listing task

A one-way ANOVA with condition (control vs. typical vs. atypical) as the independent variable and number of atypical expenses listed (i.e., expenses predicted to occur in the next week that did not occur in the past week) as the dependent variable revealed a significant effect of condition (F(2, 1,045) = 5.85, *p* = .003). Planned contrasts further indicated that the number of expenses listed in the atypical condition (M_atypical_ = 1.65, SD = 1.62) was significantly higher than in the control condition (M_control_ = 1.25, SD = 1.46; t(1,045) = 3.41, *p* < .001) and typical condition (M_typical_ = 1.39, SD = 1.59; t(1,045) = 2.14, *p* = .032) and that the number of expenses listed in the control and typical conditions did not differ significantly (t(1,045) = 1.19, *p* = .24). This provides evidence that our intervention makes atypical expenses more accessible during prediction than they would be otherwise. The same ANOVA with average dollar amount of atypical expenses as the dependent variable revealed no effect of condition (F(2, 548) = .78, *p* = .46).

#### Mediation analysis

The results show that our intervention succeeded in making atypical expenses more accessible, and that expense predictions were significantly higher in the intervention condition. To further investigate the relationship between the accessibility of atypical expenses and predictions, we tested a mediation model with condition (atypical = 1 vs. control and typical = 0) as the independent variable, expense prediction as the dependent variable, and the number of atypical expenses listed as the mediating variable. The indirect effect of condition on expense prediction via number of atypical expenses was significant (indirect effect = .04, SE = .02, CI_95%_ = [.016, .077]). Specifically, the model confirms that the atypical intervention succeeded in increasing the number of atypical expenses listed (b = .34, CI_95%_ = [.132, .548]; t(1,046) = 3.21, *p* = .001), and this number was associated with higher expense predictions even while controlling for condition (b = .13, CI_95%_ = [.089, .169]; t(1,045) = 6.28, *p* < .001).^7^

#### Perceived typicality of future expenses

A one-way ANOVA with intervention condition as the independent variable revealed a significant effect of condition on perceived typicality of future expenses (F(2, 1,044) = 32.27, *p* < .001). Planned contrasts confirmed that perceived typicality was virtually identical in the control and typical conditions (M_control_ = 4.65, CI_95%_ = [4.48, 4.81], M_typical_ = 4.65, CI_95%_ = [4.48, 4.83], t(1,044) = .03, *p* = .97), but significantly lower in the atypical condition (M_atypical_ = 3.74, CI_95%_ = [3.56, 3.93], t(1,044) = −8.02, *p* < .001, d = .55).

### Discussion

Within a nationally representative sample of U.S. consumers, Study 2 directly replicates the support we observed for H_1_–H_2_ in Study 1. This study also shows that perceived typicality and expense predictions do not change when consumers are asked to think of reasons why their future expenses might be similar to a typical week (vs. control). This is notable because it is consistent with our core proposition that consumers naturally base their predictions on their most typical expenses.

Study 2 also provides process evidence indicating that our intervention increases predictions by increasing the cognitive accessibility of atypical expenses. To further examine the effect of our intervention on the accessibility of atypical expenses and predictions, we next ran a preregistered conceptual replication (https://aspredicted.org/ni68b.pdf) in which 271 Amazon Mechanical Turk workers (35.8% female; M_age_ = 35.8 years) predicted their expenses for the next week in one of three conditions: control, typical, or atypical. The prediction instructions in each condition were the same as in Study 2, but in the replication study we also asked participants to complete the following think-aloud task as they made their prediction: “Please type every thought that enters your mind as you think about the following question and decide on your answer: How much do you estimate your total expenses will be for the next week (i.e., the next 7 days)?” After completing the think-aloud task, participants were asked to “please enter your estimated total expenses (in dollars) for the next week.”

Two research assistants who were blind to experimental condition coded the content of the think-aloud data for whether each participant referenced typical spending, future-oriented spending (i.e., atypical spending specific to the next week), or an adjustment for unexpected spending. Conceptually replicating the results of Study 2, we found that a higher proportion of participants referenced future-oriented spending in the atypical condition than in the control condition (48.3% vs. 31.8%; χ^2^(1) = 3.93, *p* = .047) or typical condition (48.3% vs. 21.0%; χ^2^(1) = 9.84, *p* = .002), but the proportion of participants in the control and typical conditions who referenced future-oriented spending did not differ significantly (χ^2^(1) = 2.10, *p* = .15). Directly replicating the results of Study 2, predictions in the atypical intervention condition (M = $393.39, CI_95%_ = [295.78, 523.22]) were 41.9% higher than in the control condition (M = $277.27, CI_95%_ = [213.79, 359.60]; t(268) = 1.90, *p* = .058), and 67.1% higher than in the typical condition (M = $235.36, CI_95%_ = [187.45, 295.48]; t(268) = 2.67, *p* = .008), but predictions in the control and typical conditions did not differ significantly (t(268) = .91, *p* = .37).

Together with Study 2, the results of this replication study provide convergent evidence that the atypical intervention increases predictions by making atypical expenses more cognitively accessible. The full “Method” section for this study and a number of other supportive results are presented as Supplemental Study A in Web Appendix F.

## Study 3: Accessibility or Diagnosticity?

Our theoretical framework proposes that the atypical intervention operates by increasing the cognitive accessibility of atypical expenses that would not otherwise come to mind during prediction. In other words, our theory implies that the intervention operates by changing prediction *content*. This is directly supported by the results of Study 2 and Supplemental Study A, and it is broadly consistent with research demonstrating that consumers often base their judgments on the substance of the information they consider (Tybout et al. 2005).

However, consistent with the accessibility-diagnosticity model (Feldman and Lynch 1988), it is possible that our intervention operates in part by increasing the diagnosticity of atypical expenses. If expense predictions are shaped by consumers’ subjective interpretations of how easy it feels to bring certain expenses to mind (Ülkümen, Thomas, and Morwitz 2008), then the simplicity of our intervention may increase prediction accuracy by leaving the impression that atypical expenses are easy to think of, creating in turn the perception that atypical expenses are relevant. In other words, the atypical intervention may work in part by changing how prediction *feels.*

To test this possibility, we adapted a classic paradigm from the diagnosticity literature to the context of expense prediction: listing few or many reasons why future expenses might be different than usual. Specifically, we had participants predict their expenses for the next week after listing zero, three, or ten reasons why their expenses might be different than usual. If predictions are driven primarily by the accessibility of atypical expenses, then listing ten reasons should lead to higher predictions than listing three reasons, because the former makes more expenses accessible than the latter. In contrast, if predictions are driven primarily by the diagnosticity of atypical expenses, then listing ten reasons should lead to lower predictions than listing three reasons, because listing ten reasons is more difficult, and prior research suggests that this could make atypical expenses feel less relevant (Tybout et al. 2005).

### Method

Seven hundred forty-nine participants completed this preregistered study (https://aspredicted.org/hg7vb.pdf) about consumer spending on Prolific Academic (55.5% female; M_age_ = 34.0 years). Participants were randomly assigned to predict their expenses for the next week in one of three conditions: the same control condition used in Studies 1 and 2 (in which no reasons were listed before prediction), or one of two intervention conditions that asked them to list either three reasons or ten reasons why their expenses for the next week might be different from a typical week before making their prediction. After making their prediction, participants in the intervention conditions rated the difficulty of the listing task using three seven-point scales anchored at “not at all difficult”/“very difficult,” “no effort”/“a lot of effort,” and “no time”/“a lot of time” (Menon and Raghubir 2003). These three items displayed a high degree of reliability (Cronbach's alpha = .87) and were combined to form a composite measure of task difficulty.

Our preregistered analysis plan for this study was contrast analyses comparing expense predictions in each condition. As per our preregistration, expense predictions were Winsorized at the 5th and 95th percentiles in each condition and natural-log transformed. As a robustness test, we also performed a nonparametric median analysis using the raw, untransformed data. That analysis produced results consistent with the following results, as detailed in Web Appendix G.

### Results

#### Manipulation check

Listing ten reasons was significantly more difficult (M = 4.72, SD = 1.47) than listing three reasons (M = 3.35, SD = 1.48), as confirmed by a between-subjects t-test (t(484) = 10.19, *p* < .001, d = .93).

#### Expense predictions

Listing three reasons why future expenses might be different than usual increased predictions by 46.9% versus listing zero reasons (M_three_ = $237.84, CI_95%_ = [$205.15, $275.72], M_zero_ = $161.87, CI_95%_ = [$144.09, $181.85]; t(746) = 3.94, *p* < .001), and listing ten reasons directionally increased predictions by 16.3% versus listing three reasons (M_ten_ = $276.50, CI_95%_ = [$237.46, $321.92]; M_three_ = $237.84, CI_95%_ = [$205.15, $275.72]; t(746) = 1.49), *p* = .14).

### Discussion

Taken together, the results of Studies 2 and 3 show that our intervention operates by increasing the accessibility of atypical expenses more than their diagnosticity. In Studies 4 and 5, we turn our attention to the relationship between typicality, predictions, and distributional skew.

## Study 4: Manipulating Skew

Our theoretical framework proposes that, in essence, consumers’ expense predictions are based on two closely related questions: (1) "What do I typically buy?" and (2) "How much do those things typically cost?" Study 4 directly tests the relevance of the second question. If our theorizing is correct, when all else is equal, a positively skewed distribution of expense amounts with mode < mean should lead to lower predictions than a normal distribution with mode = mean (H_3a_).

### Method

Four hundred participants completed this preregistered study (https://aspredicted.org/9xk89.pdf) on Prolific Academic (53.0% female; M_age_ = 33.9 years). Following André, Reinholtz, and De Langhe (2017), participants were presented with 52 weekly expense amounts, one at a time, every 1.2 seconds. The 52 expense amounts were drawn in random order without replacement from either a normal distribution with mean = median = mode = $200, SD = 18.89, and skew = .00, or a positively skewed distribution with mean = median = $200, mode = $190, SD = 18.89, and skew = .29. (For histograms of the distributions used in this study and its replicate, see Web Appendix H.) After viewing all 52 values from either the normal or skewed distribution, participants were asked to predict their expense amount for the next week.

Our preregistered analysis plan for this study was (1) an independent samples t-test comparing predictions between the two conditions, (2) one-sample t-tests comparing predictions in each condition to $200, and (3) a chi-squared test of proportions measuring the percentage of participants in each condition who made predictions less than the $200 mean of the distributions we showed them. As per our preregistration, predictions were Winsorized at the 5th and 95th percentiles of each condition for analyses (1) and (2).

### Results

#### Expense predictions

The amount consumers predicted they would spend in the next week was significantly lower in the skew condition (M = $191.41, SD = 29.73) than in the normal distribution condition (M = $198.00, SD = 14.09), as revealed by an independent samples t-test (t(398) = 2.85, *p* = .005, d = .29). One-sample t-tests further revealed that predictions in the skew condition were significantly lower than the $200 mean of the underlying distribution (t(196) = −4.06, *p* < .001) but not significantly different than the $190 mode (t(196) = .66, *p* = .51). One sample t-tests also revealed that predictions in the normal condition were slightly lower than $200 (t(202) = −2.02, *p* = .044), but a great deal higher than $190 (t(202) = 8.09, *p* < .001). Taken together, this pattern of results shows that a positively skewed distribution with mode < mean led to lower predictions than a normal distribution with mode = mean, all else equal.

#### Distribution of predictions

The proportion of participants who predicted spending less than $200 was significantly higher in the skew condition than in the normal distribution condition (31.0% in the skew condition vs. 18.7% in the normal condition; χ^2^(1) = 8.10, *p* = .004). Moreover, the proportion of participants who predicted spending equal to $200 was significantly lower in the skew condition than in the normal condition (50.8% in the skew condition vs. 64.0% in the normal condition; χ^2^(1) = 7.11, *p* = .008), but the proportion of participants who predicted spending more than $200 was very similar in both conditions (18.3% in the skew condition vs. 17.2% in the normal condition; χ^2^(1) = .08, *p* = .77). This pattern of results indicates that the skewed distribution shifted predictions away from the mean and toward the mode.

### Discussion

Study 4 provides support for H_3a_ by showing that when all else is equal, the amount consumers predict they will spend is lower and farther away from the mean when the distribution of expense amounts is positively skewed than normal distributed. This finding is replicated in Supplemental Study B in Web Appendix H. We view this finding as a fairly conservative estimate of the relationship between skew and predictions because participants were presented with the full distribution of past outcomes—including atypical outcomes—right before predicting. We next examine the relationship between our intervention and predictions in real-world expense categories that naturally display different levels of skewness.

## Study 5: Intervention Effectiveness and Skewness “in the Wild”

We conducted Study 5 in partnership with Money Dashboard (MDB), a popular personal finance app in the United Kingdom (https://www.moneydashboard.com/). The goal of this study was to examine the relationship between the atypical intervention and predictions in expense categories that naturally display different degrees of skewness. The logic underlying the atypical intervention is that when expenses are positively skewed, thinking of reasons why expenses will be different than usual means thinking of individual expenses that make total weekly spending higher than usual. However, when expenses are more normally distributed, “different than usual” means “higher than usual” or “lower than usual” with equal probability. This implies that our intervention should exert less influence on predictions when the distribution of expenses is less skewed, because in a more normal distribution the outcomes that are different than the mode are often lower than the mode. This logic can be visualized by revisiting Figure 1. In the positively skewed distribution on the right, “different than usual” mostly means “higher than usual.” But in the normal distribution on the left, “different than usual” means “higher than usual” or “lower than usual” with equal probability. Therefore, our expectation in this study was that expense category skewness would act as a boundary condition of the atypical intervention, such that the intervention increases predictions in a real-world expense category with relatively strong positive skew more than in a category with relatively moderate positive skew (H_4_).

### Method

#### Participants and procedure

Participants in this preregistered field experiment (https://aspredicted.org/jv9hg.pdf) were consumers who use the personal finance app MDB. The link to the experiment was emailed to MDB users (approximately 100,000 U.K. residents) within their monthly newsletter. Specifically, the newsletter advertised a ten-minute consumer finance study that could be completed in exchange for the opportunity to win one of five £1,000 cash prizes. As per our preregistration, the study link was active for one week. By the end of the week 1,738 MDB users had completed the study (24.1% female; M_age_ = 41.6 years).

Participants were randomly assigned to predict their expenses for the next week (i.e., the next seven days) in a 2 (condition: control vs. atypical) × 2 (category: online shopping vs. grocery shopping) × 2 (expenses: predicted vs. actual) between-within study design, in which condition and category were manipulated between-subjects, and expenses were measured within-subject. The control and atypical prediction instructions were the same as in Study 1 but with two changes. First, the instructions in both prediction conditions were worded to refer to either online shopping, which we defined for participants as “all purchases you make through an app or website,” or grocery shopping, which we defined as “all purchases you make at the grocery store.” We chose online and grocery shopping as the prediction categories for this study because average within-subject skewness for weekly online shopping (.98) was significantly higher than average within-subject skewness for weekly grocery shopping (.55) over the 20 weeks preceding the study (t(1,734) = 17.68, *p* < .001), and because these were the two most common types of expenses incurred by app users during that time frame. Second, the atypical intervention in this study asked participants to “please type at least one reason why your online [grocery] expenses for next week might be *different* from a typical week” rather than asking them to type three reasons, as in Studies 1–3. We made this change because we wanted to make the intervention as simple as possible for consumers to complete in a field setting, and a pretest indicated that listing one reason is sufficient to increase predictions. After participants made their predictions, their online and grocery spending was tracked through the app for one week so that we could measure their prediction accuracy. As per our preregistration, expenses were natural log–transformed for inferential analyses.

### Results

**Figure 5.**
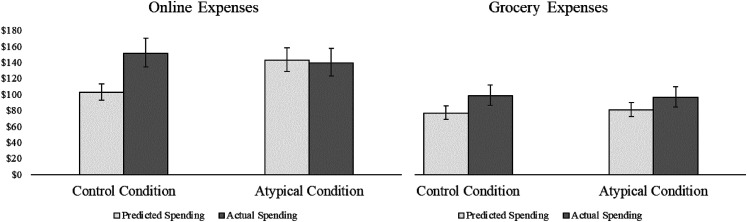
Mean Predicted and Actual Expenses in Each Condition of Study 5.

The results of Study 5 are illustrated in Figure 5. A 2 (condition: control vs. atypical) × 2 (category: online vs. grocery) × 2 (expenses: predicted vs. actual) mixed-model ANOVA revealed a significant main effect of category (F(1, 1,734) = 77.51, *p* < .001, $\eta_{p}^{2}$ = .04), a significant main effect of expenses (F(1, 1,734) = 30.10, *p* < .001, $\eta_{p}^{2}$ = .02), a significant two-way interaction between expenses and condition (F(1, 1,734) = 11.17, *p* < .001, $\eta_{p}^{2}$ = .01), and a significant three-way interaction between expenses, condition, and category (F(1, 1,734) = 5.62, *p* = .018, $\eta_{p}^{2}$ = .003). No other omnibus effects were significant (*p*s ≥ .14). Participants who predicted their online expenses in the control condition underpredicted by £48.65 (32.2%), as compared with the online expenses they actually incurred during the week after prediction (t(478) = −4.64, *p* < .001). However, participants who predicted their online expenses in the atypical condition were very accurate: their predicted expenses differed from their actual expenses by only £3.10 or 2.2% (t(451) = .28, *p* = .78). Online expense prediction accuracy differed between the control and atypical condition because predictions were 39.2% higher in the atypical condition (M = £142.45, CI_95%_ = [128.51, 157.91]) than in the control condition (M = £102.31, CI_95%_ = [92.57, 113.07]; t(929) = 3.70, *p* < .001), but actual online expenses in the atypical condition (M = £139.35, CI_95%_ = [123.22, 157.59]) did not differ significantly from actual online expenses in the control (M = £150.96, CI_95%_ = [134.29, 170.20]; t(929) = −.86, *p* = .39). This pattern of results replicates the effect of the atypical intervention on prediction accuracy (vs. control) that we observed for total weekly spending in Study 1.

Participants who predicted their grocery expenses in the control condition underpredicted by £21.44 or 21.8% (t(420) = −4.46, *p* < .001), and participants who predicted their grocery expenses in the atypical condition underpredicted by £15.52 or 16.1% (t(385) = −3.01, *p* = .003). The size of the prediction bias did not differ significantly between the two conditions (t(805) = .87, *p* = .38). As we expected, predicted grocery expenses were only slightly higher in the atypical condition than in the control (M_atypical_ = £80.64, CI_95%_ = [72.17, 90.20]; M_control_ = £76.86, CI_95%_ = [69.06, 85.54]; t(805) = .93, *p* = .35), and actual grocery expenses in each condition were almost equivalent (M_atypical_ = £96.16, CI_95%_ = [84.18, 109.84]; M_control_ = £98.30, CI_95%_ = [86.49, 111.61]; t(805) = .25, *p* = .80). These results support our hypothesis that the atypical intervention exerts less influence on predictions when expenses are less positively skewed (H_4_).

Finally, participants who predicted their online expenses in the control condition were significantly less accurate than participants who predicted their grocery expenses (t(1,284) = 2.13, *p* = .033). This conceptually replicates the results of Study 4 using real world expense categories that naturally differ in the amount of skewness they display (H_3b_), demonstrating the critical role of skew in consumer prediction accuracy for real-world expenses, measured objectively.

#### Robustness tests

As noted previously, we chose the expense categories in this study because they displayed different levels of within-subject skew over the 20 weeks preceding the study. However, they also displayed different weekly means (online = £134.10 vs. groceries = £82.11; t(1,736)= 9.71, *p* < .001), standard deviations (online = 174.88 vs. groceries = 57.54; t(1,736) = 22.79, *p* < .001), and ranges (online = £674.86 vs. groceries = £209.77; t(1,736) = 22.67, *p* < .001). We therefore performed each of the preceding analyses using these variables as controls. The three-way interaction between condition, category, and expenses was not meaningfully affected by the inclusion of these control variables (F(1, 1,731) = 7.74, *p* = .005), nor were the contrasts comparing predicted and actual expenses. Web Appendix I presents the detailed results of this analysis, along with the results of a winsorized mean and nonparametric median analysis.

#### Discussion

Study 5 conceptually replicates the results of Study 4 using real-world expense categories that naturally differ in terms of skew (H_3b_), and it identifies skewness as a boundary condition of the atypical intervention's effect on predictions (H_4_). The strengths of working with this kind of real-world data include their scope and accuracy; one limitation is that the expense categories differ in terms of more than skew. Nonetheless, we observe the expected pattern of results even when we control for mean, standard deviation, and range, and the results stand up to the robustness checks presented in Web Appendix I.

## General Discussion

The present research provides evidence that consumers display an expense prediction bias in which they significantly underpredict their future expenses. This bias occurs in part because consumers base their predictions on typical expenses (e.g., groceries) and typical expense amounts (e.g., $150 per week) that come to mind easily (think-aloud pilot study, Study 2, Supplemental Study A). Taken together, predictions based on typical expenses are closer to the *mode* of a consumer's expense distribution than the mean (Studies 1 and 4, and Supplemental Study B). This leads to underprediction because, generally speaking, the distribution of expenses is positively skewed with mode < mean (Studies 1 and 5). However, consistent with our proposition that predictions are based on typical expenses that constitute modal spending, we find that predictions are closer to the mean when expenses are more normally distributed (Studies 4 and 5, and Supplemental Study B).

Under the general case of positively skewed expenses, we find that prompting consumers to consider reasons why their expenses might be *different* than usual increases predictions—and therefore prediction accuracy—by bringing atypical expenses to mind (Studies 1, 2, 3, and 5; Supplemental Studies A, C, and D). However, supporting our theory, this “atypical” intervention is less effective when expenses are more normally distributed (Study 5) and “different than usual” represents reasons why expenses might be higher or lower with more equal probability. It is also notable that we do not find statistically significant support for the possibility that the atypical intervention operates by altering the diagnosticity of atypical expenses, nor do we observe a significant correlation between the bias and individual differences such as prediction confidence, trait optimism, or the presence of a savings goal. Finally, because our studies in the main text focus on weekly predictions, we note here that Supplemental Studies C and D (in Web Appendices J and K, respectively) demonstrate that our theory can be applied to monthly predictions as well. We next discuss our findings in relation to relevant previous research.

### Cognitive Accessibility and the Psychology of Prediction

The dominant theoretical perspective regarding the psychology of prediction is that prediction biases occur in large part because people fail to incorporate relevant past experience when predicting the future. This perspective has been invoked in research on the planning fallacy (Buehler, Griffin, and Ross 1994), affective forecasting (Wilson et al. 2000), prosocial behavior (Epley and Dunning 2000), and financial decision making (Peetz and Buehler 2012). However, our findings indicate that expense predictions are in fact largely based on typical expenses representing highly relevant past behavior, and that this is exactly why predictions are biased. We believe our findings can be reconciled with research in other domains by considering what information is most cognitively accessible during each type of prediction task. In the case of expenses, the most accessible information is a consumer's typical past expenses, which are easily learned and remembered because they are reinforced by frequent purchasing behavior. However, in the case of project planning, for example, there may not be the same volume of often-repeated behavior for an individual to draw from. Therefore, their most accessible information during prediction is likely to be what they envision achieving in the future rather than what they have actually achieved in the past (Kahneman and Tversky 1979).

In addition to demonstrating *why* consumers underpredict their future expenses, our research also shows *how* prediction accuracy can be improved. Mechanistically, our “atypical intervention” bears some resemblance to the unpacking intervention derived from support theory (Kruger and Evans 2004), in which people are asked to “unpack” their prediction into its component parts (e.g., individual expenses) to elicit broader consideration of possible future outcomes (Peetz et al. 2015). There is, however, an important distinction between our intervention and unpacking: the latter prompts people to consider all possible outcomes, while the former prompts them to consider only atypical outcomes. This is important from a theoretical perspective because the unpacking intervention says only that distributional information is missing from predictions. In contrast, the atypical intervention deepens our understanding of *which* distributional information is missing. The atypical intervention also carries a practical advantage: it only requires considering a small number of reasons why expenses may be atypical (vs. trying to unpack all possible expenses), which makes it easier to employ. This is noteworthy given that expense predictions are often made spontaneously (Peetz et al. 2016), which suggests that a simpler tool will be more widely used.

#### Typicality

The present research also advances knowledge about the use of “prototype attributes” in judgment and decision making. A prototype attribute is a mental representation of what a person perceives to be typical or average (Kahneman 2003; Kahneman and Frederick 2002). In the context of expenses, this includes the individual expenses a consumer most typically purchases (e.g., groceries) and the amount each of these expenses typically costs them (e.g., $200). While prior research offers reasons to believe that typical expenses could represent the *mean* of a consumer's expense distribution (André, Reinholtz, and De Langhe 2017; Beach and Swenson 1966), the present research indicates that in the case of expense predictions, prototype attributes represent outcomes that more closely track the *mode* of a distribution.

#### Skewness

Previous research on prediction accuracy has acknowledged the detrimental impact that underweighting or ignoring distributional information can have on prediction accuracy (Buehler, Griffin, and Peetz 2010). However, the potential impact of distributional skew on prediction accuracy has received very little attention in this literature. Therefore, one novel aspect of our theory is that it uses skewness to predict *when* consumers will show more or less bias in their predictions, and when the atypical intervention will be more or less effective.

### Implications for Consumers and Firms

An important contribution of the present research is that it provides a more comprehensive understanding of expense prediction bias as a phenomenon by documenting its magnitude, persistence, and prevalence in nonstudent samples. Our studies are also the first to examine the bias longitudinally and in the field, and to measure monthly expense prediction accuracy. The implications of our findings in this regard are clear: the magnitude of the bias—approximately $100/week or $400/month in Study 1—is large enough to be economically meaningful for many consumers. Thus, the prosocial benefit of our research is also clear—any consumer can use the atypical intervention to improve their expense prediction accuracy and make better informed decisions regarding their spending, borrowing, or saving behavior.

One promising channel through which the atypical intervention can be applied is financial literacy organizations. Currently, the standard approach in these organizations is to educate their stakeholders about debits and credits, interest rates, and so on. However, this approach is time consuming and has limited impact (Fernandes, Lynch, and Netemeyer 2014). In contrast, the atypical intervention can be easily provided and used to effectively increase prediction accuracy.

Our findings also have practical implications for for-profit firms. For example, companies in the FinTech sector that develop budgeting apps can leverage our results to design their products in a way that helps users set more realistic budgets. Given that 63% of North Americans with a smartphone have at least one financial app on their phone (Barba 2018)—the key function of which is often budgeting—this could confer a substantial product advantage. Furthermore, because many behaviors likely follow a skewed distribution, we believe that the atypical intervention can also be used to inform the design of products that aim to improve consumers’ predictions and plans with respect to calories, exercise, sleep, and a host of other variables that can positively impact consumers’ well-being.

### Limitations and Directions for Future Research

One limitation of the present research is that it investigates the relationship between typical outcomes, skewness, and predictions in a single domain. Therefore, an important direction for future research is to determine the generalizability of our theoretical framework beyond expense prediction. Previous research has concluded that individuals are quite adept at mean-identification with respect to sets of shapes (Ariely 2001), numbers (André, Reinholtz, and De Langhe 2017), and faces (Haberman and Whitney 2009). However, it is not clear that the distributions of stimuli in these studies allowed participants to differentiate between the mode and mean. This raises the intriguing possibility that what people perceive to be typical or average in these contexts could be the mode, as our research suggests is the case for expenses. Future research should also examine the perceptual difference between modal and median outcomes and test the possibility that the latter also influences perceived typicality.

A second limitation of the present research is that it tests one specific method of making atypical expenses more accessible. This approach lets us establish that the intervention is robust to scenario (field and lab), sample (app users, credit union members, Mechanical Turk workers, and students), and time frame (week and month), but it is important for future research to examine different methods of making atypical expenses accessible. Prior research shows that people are more accurate when predicting for others than for themselves (e.g., Buehler, Griffin, and Ross 1994), so one way to accomplish this could be to prompt consumers to take a third-party perspective when predicting. Interventions such as “dialectic bootstrapping” (Herzog and Hertwig 2009) and an taking an “outside view” (Kahneman and Tversky 1979) could also make atypical expenses more accessible, though the level of involvement they require may make them difficult to implement in practice.

Finally, whereas the present research focuses on factors that affect predictions, future research should focus on the link between predictions and downstream behaviors such as decisions to spend, save, or borrow. Supplemental Study D finds that the atypical intervention increases intentions to save in addition to expense predictions (for details, see Web Appendix K). An important next step for research in this area is to time the intervention to coincide with specific financial decisions and measure its ability to influence downstream behaviors.

## Supplemental Material

sj-pdf-1-mrj-10.1177_00222437211068025 - Supplemental material for Understanding and Neutralizing the Expense Prediction Bias: The Role of Accessibility, Typicality, and Skewness Supplemental material, sj-pdf-1-mrj-10.1177_00222437211068025 for Understanding and Neutralizing the Expense Prediction Bias: The Role of Accessibility, Typicality, and Skewness by Ray Charles “Chuck” Howard, David J. Hardisty and Abigail B. Sussman, Marcel F. Lukas in Journal of Marketing Research

## Notes

1 We use the term “mode” as shorthand for the peak of a smoothed probability distribution function, or, analogously, the most common value in a binned histogram. In reality, a consumer’s distribution of expenses is continuous (e.g., $149.69, $150.21, $159.27). However, it is reasonable to assume that, in a consumer’s memory, their distribution of expenses consists of round, discrete values (e.g., $150, $150, $160), because consumers are inattentive to decimal places (Bizer and Schindler 2005) and they automatically categorize information in approximate ways (Harnad 2017). This assumption is also supported by our study results: virtually all participants estimate expenses using round numbers (e.g., Table 1). Although we focus on the mode, it is also possible that predictions fall near the median. We examine this in Study 4.

2 We focus predominately on weekly expense predictions because research suggests that they are more common than monthly predictions (Peetz et al. 2016). However, Study 1 shows that consumers underpredict their monthly expenses as well, and Supplemental Studies C and D show that our theoretical framework also applies to monthly expense predictions.

3 In this article, we operationalize an “expense” as a single expense transaction (e.g., a credit card charge). For example, going to a restaurant and buying lunch would most often be mentally estimated as a single expense (“lunch”) rather than a disaggregated series of subcomponent expenses (“burger, cheese, fries, a drink, and sales tax”).

4 Participant dropout over the last week of the study was minimal (n = 4) and did not differ by condition.

5 A set of 2 (order: predict first vs. recall first) × 3 (condition: control vs. typical vs. atypical) ANOVAs with predicted expenses, recalled expenses, and bias scores (recalled − predicted expenses) as the dependent variables revealed no order effect (*p*s > .17).

6 As mentioned in footnote 5, the order of prediction and recall does not interact with condition, but adding order to the model does reveal directionally lower recall for participants in the typical condition who predicted first, and directionally higher recall for participants in the atypical condition who predicted first. Therefore, the difference in recalled expenses between these two conditions may be the result of the prediction manipulation in each condition spilling over into recall.

7 The same results are obtained when using only the atypical and pure control conditions as levels of the independent variable (indirect effect = .06, SE = .02, 95% CI = [.02, .09]) and when running a categorical mediation model that includes all three conditions (indirect effect of atypical dummy = .05, SE = .02, 95% = [.02, .08]; indirect effect of typical dummy = −.01, SE = .02, 95% CI = [−.04, .02]).
