# Supplementary material for: Understanding and Neutralizing the Expense Prediction Bias: The Role of Accessibility, Typicality, and Skewness
Source: J Mark Res. 2022 Feb 15;59(2):435–52. doi: 10.1177/00222437211068025 (PMC13038220; doi:10.1177/00222437211068025)
Supplement: sj-pdf-1-mrj-10.1177_00222437211068025 - Supplemental material for Understanding and Neutralizing the Expense Prediction Bias: The Role of Accessibility, Typicality, and Skewness [file sj-pdf-1-mrj-10.1177_00222437211068025.pdf]

WEB APPENDIX

UNDERSTANDING AND NEUTRALIZING THE EXPENSE PREDICTION BIAS:

THE ROLE OF ACCESSIBILITY, TYPICALITY, AND SKEWNESS

Ray Charles "Chuck" Howard
Assistant Professor of Marketing, Mays Business School, Texas A&M University
rhoward@mays.tamu.edu

David J. Hardisty
Associate Professor of Marketing, Sauder School of Business, University of British Columbia
david.hardisty@sauder.ubc.ca

Abigail B. Sussman
Associate Professor of Marketing, Booth School of Business, University of Chicago
abigail.sussman@chicagobooth.edu

Marcel F. Lukas
Lecturer in Banking and Finance, School of Management, University of St. Andrews
mfl3@st-andrews.ac.uk

Please direct correspondence to Chuck Howard (rhoward@mays.tamu.edu). This work was funded by the Social Sciences and Humanities Research Council of Canada, True North Communications Inc. Faculty Research Funds at The University of Chicago Booth School of Business, and the Mays Business School Dean's Office. For insightful feedback, the authors thank Dale Griffin, John Lynch, Leaf Van Boven, Elizabeth Dunn, the Editor, Associate Editor, and three anonymous reviewers. For outstanding research assistance, the authors thank Andrew Lai, Alena Goncharova, Kelly Yang, Luc Parlange, Kavita Dau, Tiffany Leung, and Larissa Franco. Supplementary materials are included in the web appendix accompanying the online version of this article. Data, syntax, and preregistration files are available on the Open Science Framework: https://osf.io/k26ng/?view_only=bc72f72dc985460a9fc6c9e6f2f9e7b9.

"These materials have been supplied by the authors to aid in the understanding of their paper. The AMA is sharing these materials at the request of the authors."

TABLE OF CONTENTS

WEB APPENDIX A: TOP-DOWN PREDICTION 2

WEB APPENDIX B: DATA COLLECTION DETAILS AND ANALYSIS OF EXPLORATORY VARIABLES IN STUDY 1 4

WEB APPENDIX C: ROBUSTNESS TESTS FOR STUDIES 1 AND 2 9

WEB APPENDIX D: DATA TRANSFORMATION PROCESS IN STUDIES 1 AND 2 18

WEB APPENDIX E: ANALYSIS OF EXPLORATORY VARIABLES IN STUDY 2 20

WEB APPENDIX F: DETAILS OF SUPPLEMENTAL STUDY A 23

WEB APPENDIX G: NON-PARAMETRIC ANALYSIS FOR STUDY 3 28

WEB APPENDIX H: RESULTS OF SUPPLEMENTAL STUDY B 29

WEB APPENDIX I: STUDY 5 ROBUSTNESS TESTS 31

WEB APPENDIX J: RESULTS OF SUPPLEMENTAL STUDY C 35

WEB APPENDIX K: RESULTS OF SUPPLEMENTAL STUDY D 38

WEB APPENDIX A: TOP-DOWN PREDICTION

The main manuscript explores expense prediction, the roles of "typical" and "atypical" expenses, and the importance of skew, all in the context of "bottom-up" prediction. However, according to our survey study, some people report using a "top-down" approach to prediction. Here we explain how our theory applies to top-down prediction.

Imagine Tiffany, who monitors her credit card bill and bank account balance, but doesn't track individual expenses very closely. She might know that most frequently, she spends about $500 each week. Therefore, when Tiffany wants to predict her total expenses for the next week, she might take a "top-down" approach, and directly predict $500 (the amount she typically spends each week). In other words, Tiffany directly predicts the "mode" of her total weekly spending.

Note that by predicting the mode, Tiffany's prediction will often be accurate, by definition. However, as shown in Figure 1 of the main manuscript, her predictions can still be biased, depending on the shape of her expense distribution. If her expenses are normally distributed, she will be unbiased; sometimes she will over-predict, and sometimes she will underpredict, but these errors will be equal in frequency and magnitude. However, if the distribution of her weekly expenses is positively skewed, she will systematically underpredict her mean expenses over time. This is because she will occasionally make large errors of underprediction, and will occasionally make small errors of overprediction; because her errors of underprediction will be larger, she is biased in her predictions. Depending on the shape of the distribution, she may also more *frequently* under-predict than over-predict her expenses, if the mode of the distribution is lower than the *median*. In summary, a top-down approach to prediction when expenses are positively skewed with mode < mean will always lead to a bias in

the *magnitude* of errors, and may also lead to a bias in the *frequency* of errors (if mode < median).

Note that the process outlined above concerns the generation of an initial prediction (i.e., “stage 1” prediction). Sometimes this is followed by a buffer adjustment (i.e., “stage 2” prediction). For example, Tiffany may notice that she sometimes underpredicts her expenses, so after predicting her most frequent weekly expense amount she may add a buffer amount, making her final prediction somewhat higher than the mode. However, even with this buffer, her prediction is still likely to be lower than the mean because stage 2 adjustments tend to be too small to prevent underprediction (Ulkumen et al. 2008).

WEB APPENDIX B:

DATA COLLECTION DETAILS
AND ANALYSIS OF EXPLORATORY VARIABLES IN STUDY 1

Data Collection Details

In wave 1, we sent a survey at time zero (T0) to 400 randomly selected members at noon on a Sunday. Ninety-three people completed the survey before it was deactivated at 11:59pm the next day. We then monitored attrition for two weeks before calculating that the T0 survey should be sent to another 800 randomly selected members (the maximum number allowed by our field partner) in the second wave of data collection so that we could recruit as close to 200 total participants as possible. In wave 2, 219 members completed the T0 survey. At the end of both waves of data collection we had complete data from 187 participants (61 from wave 1 and 126 from wave 2, M_{age} = 51.12, 57.8% female).

Analysis of Exploratory Variables

Study 1 explored the relationship between EPB and several theoretically and practically relevant individual differences that may be predictive of the bias. The first was the presence of a savings goal, because motivation to save has been tied to lower predictions (Peetz and Buehler 2009, 2013). The second was trait optimism (Scheier, Carver, and Bridges, 1994). We predicted that trait optimism would actually *not* be correlated with EPB because research on the planning fallacy has demonstrated that optimistic task completion time predictions are not the result of an optimistic disposition (Buehler, Griffin, and Ross, 1994). Nonetheless, because the relationship

between trait optimism and expense predictions seems intuitively compelling and has not yet been explored, we felt there was value in testing it.

The third individual difference we measured was short-term financial propensity to plan (PTP; Lynch et al., 2010). One prediction regarding the relationship between short-term financial PTP and EPB is that consumers with a higher PTP will display lower EPB because they are more attuned to (or concerned with) future outcomes. However, it could be the case that greater plan-focus leads consumers to be less attentive to unplanned expenses and therefore more likely to under-predict their future expenses (cf. Buehler, Griffin, and Peetz, 2010). We therefore included the Lynch et al. (2010) measure of short-term financial propensity to plan so that we could explore these competing hypotheses.

The fourth individual difference measure we included was the Rick, Cryder, and Lowenstein (2008) spendthrift-tightwad scale. There are three hypotheses for how this measure could be correlated with EPB. The first hypothesis is that tightwads may display *higher* EPB because anticipatory pain of paying causes them to predict even lower expenses than they actually incur. The second hypothesis is that tightwads could display *lower* EPB if they are more sensitive to expenses and therefore more accurate. Finally, spendthrifts may display higher EPB because they lack pain of paying during purchase and therefore may be more likely to overspend vs. prediction.

The fifth individual difference measure we included was numeracy (Schwartz et al., 1997) because consumers who are unable to perform the mental calculations required to make an accurate expense prediction may display higher EPB. The sixth measure was linear vs. cyclical time orientation (adapted from Tam and Dholakia, 2014). Consumers with a stronger cyclical orientation may display lower EPB because they see life events as a series of recurring events

(Tam and Dholakia, 2014) and therefore should be more easily able to incorporate past atypical expenses into their predictions for the future. The seventh individual difference we included was openness to experience (John, Donahue, and Kentle, 1991), because being more likely to consider a wider range of outcomes when making predictions could be associated with lower EPB. We also measured temporal discounting for both losses and gains (Kirby & Maraković 1996). Consumers with a relatively high discount rate for losses (i.e., those with a stronger preference to pay more later vs. less now) may display higher EPB because they may want to postpone payment as much as possible, and our measure of EPB in this study was for the coming the week. The same logic applied in reverse led us to belief that temporal discounting of gains may be negatively correlated with EPB.

In addition to the individual differences of theoretical interest above, we also included measures that let us explore the relationship between EPB and preferred form of payment (credit card, debit card, cash, other), budgeting behavior, perceived expense predictability, socio-economic status, gender, and education. We reasoned that heavier reliance on cards as a form of payment might be correlated with EPB because using credit cards reduces pain of payment (Thomas, Desai, & Seenivasan, 2011), which could lead to spending more than predicted. Budgeting could lead to lower EPB by prompting consumers to consider a wide array of future outcomes, or higher EPB by causing them to focus on minimizing predicted (but not actual) expenses in pursuit of a savings goal (Peetz and Buehler, 2009). We expected that higher perceived predictability could be correlated with higher EPB, because it may be associated with higher prediction confidence, which has been shown to lead to less accurate predictions (Ülkümen et al., 2008). Finally, we wanted to explore the relationship between EPB and socio-economic status (SES), and EPB and education, to determine if neutralizing the bias could be of

particular benefit to vulnerable consumers (i.e., those who are low SES or who have less formal education).

As can be seen in Table W1, our exploration of the relationship between EPB and the variables described above yielded mostly null results. One explanation for this could be a lack of power – our sample size was determined by a power analysis that included an estimate of the EPB effect size, not an estimate of the correlation between EPB and these measures. Of course, a second explanation is that there is no meaningful relationship between EPB and these measures. In our view, this possibility is perhaps most exciting, because it suggests that typical prediction is a general cognitive process that causes expense under-prediction regardless of a consumer's orientation with respect to the individual difference variables we measured. However, future research is required to test this conjecture.

Table W1
Individual Difference Null Results in Study 1

	Correlation between Individual Difference Measures and EPB in Study 1										
	Savings Goal	Trait Optimism	Propensity to Plan	Numeracy	Spendthrift - Tightwad	Openness to Experience	Temporal Discounting (Losses)	Temporal Discounting (Gains)	Risk Aversion (Losses)	Risk Aversion (Gains)	Cyclical vs. Linear Time Orientation
Week 1 EPB	-0.06	0.03	-0.03	0.00	-0.05	0.09	0.08	-0.02	0.13	-0.06	0.06
Week 2 EPB	-0.15*	0.04	-0.01	0.08	-0.09	-0.09	-0.09	-0.07	0.00	-0.07	0.01
Week 3 EPB	0.03	0.07	0.03	0.01	0.00	0.11	-0.04	0.03	0.00	0.06	0.04
Week 4 EPB	0.09	0.05	0.15*	-.07	0.04	0.11	-0.06	-0.06	0.04	0.02	-0.03
Monthly EPB	0.02	0.09	0.08	-0.05	0.07	0.10	0.14	-0.09	0.01	0.04	0.00

*p < .05

Correlation between Individual Difference Measures and EPB in Study 1 (Continued)											
	Credit Card	Debit Card	Cash	Sets Budget Y/N	Time Spent Budgeting	Expense Predictability	LN(Available Resources)	SES	Annual Income	Gender (male=1; female=0)	Education
Week 1 EPB	-0.06	0.08	-0.04	-0.09	0.02	-0.07	0.03	0.00	0.09	-0.12	-0.04
Week 2 EPB	0.02	-0.01	-0.05	-0.11	0.13	0.03	0.11	0.02	0.07	0.12	0.12
Week 3 EPB	-0.05	0.02	0.09	0.04	0.05	0.07	-0.10	0.09	0.04	0.09	0.05
Week 4 EPB	-0.04	0.01	0.02	0.13	0.02	0.03	-0.08	-0.08	-0.01	0.01	0.06
Monthly EPB	0.01	-0.05	0.02	-0.03	0.00	0.04	0.01	-0.03	-0.08	-0.13	0.11

*p < .05

We also used Study 1 to explore the relationship between EPB and potential correlates of the bias such as savings, debt, and subjective financial well-being. As illustrated in Table W2, this exploration also yielded mostly null results. As noted above, this could be due to a lack of

power or a truly null relationship. It could also be due to measurement error: it is possible that not all expenses were posted online when participants reported their expenses at the end of each week, so our measure of EPB might be somewhat conservative, which could be suppressing the link between EPB and these measures. It is also possible that it is persistence in monthly EPB that links under-prediction with these variables, which would mean we were unable to capture relationships using (predominately) weekly measures of EPB. Examining these possibilities falls well outside the scope of this article, but certainly warrants examination in future research.

Table W2

Null Results for Potential Bias Correlates Measured in Study 1

	$1,000 Allocation Decision Task			Savings		Debt		Subjective Financial Measures			
	Save	Repay Debt	Spend	LN(Target Monthly Savings)	LN(Actual Monthly Savings)	Fair & Fast Loan Use (Y/N)	Borrows from Friends and Family	Financial Well-Being	Financial Security	Financial Slack	Spends more than expected
Week 1 EPB	.12	-.13	.03	.01	.10	.09	.01	.08	.04	.02	-.06
Week 2 EPB	-.05	.09	.16*	-.05	-.06	-.02	-.02	-.01	-.07	-.05	.06
Week 3 EPB	-.03	-.02	.14	.13	.09	.01	-.05	.04	0.13	-.07	-.06
Week 4 EPB	-.03	.02	-.02	-.05	-.03	-.11	-.05	-.03	-.03	-.03	-.05
Monthly EPB	.01	.07	-.02	-.14	-.05	-.07	-.13	.03	.01	-.07	-.08

WEB APPENDIX C:

ROBUSTNESS TESTS FOR STUDIES 1 AND 2

The distribution of consumer expenses displays strong positive skew, which presents serious challenges for responsible inferential analysis. In Studies 1 and 2 we addressed this by excluding the data of participants whose reported expenses exceed their predicted expenses by more than a factor of 10 (or vice versa), then LN-transforming the distributions of reported and predicted expenses. The first advantage of this approach is that it allows us to analyze (and visualize) EPB as the difference between reported and predicted expenses. In contrast, if we adopted an approach like winsorization we could only present winsorized mean bias scores, because winsorizing reported and predicted expenses independently could erase or even reverse some bias scores.[1] Therefore, a clear drawback of winsorization is that presenting only bias scores means the reader has no way of knowing if the bias occurred (or not) because of the effect of our atypical intervention on predictions, or because of variation in reported expenses between conditions.

The second advantage of the approach we use in the main text is that once results are exponentiated into dollars they are very easy to interpret, regardless of a reader's background in statistics. In contrast, taking a non-parametric approach that analyzes the median difference between reported and predicted expenses yields results that can be counterintuitive for readers who are unfamiliar with these methods. For example, if the median of reported expenses is $180

---

[1] To illustrate this point, imagine a participant who reports expenses of $800, predicts expenses of $700, and therefore has a bias score of $100. If the dollar value at the 95th percentile of reported and predicted expenses is equivalent and <= $700, then this participant's bias score will become zero. Also consider a scenario where the 95th percentile of reported expenses is $690, and the 95th percentile of predicted expenses is $700. In this case winsorizing reported and predicted expenses independently would reverse this participant's bias score from $100 to -$10.

and the median of predicted expenses is \$150, the median *difference* between reported and predicted expenses will quite likely *not* equal \$30.00. Issues such as this are further compounded in multi-condition studies where condition is a between-subjects variable and expenses (reported vs. predicted) are measured within-subject.

Despite the drawbacks of winsorization and non-parametric analysis, we feel it is important to present them here as robustness tests. In this appendix we also present the non-parametric correlations between perceived typicality and predicted expenses (H1b), and we reperform our parametric tests of H1a to show that the results do not change when there are no data exclusions. (All analyses performed for this appendix were performed on the full data, with no data exclusions.) Finally, in Web Appendix D, we detail the impact that each step of our transformation process has with respect to the magnitude of the bias, homogeneity of variance between conditions and time periods, and normality.

STUDY 1

Weekly Expense Prediction Bias (Recalled minus Predicted Expenses). As illustrated in Figure W1 and Table W3, there was a significant bias in each week of the study until week 5 when the atypical intervention effectively eliminated the bias, as measured by either the winsorized mean bias score or raw median bias score. A pair of Mann-Whitney U tests further reveal that the median prediction in the atypical condition was higher than in the control ($\text{Median}_{\text{atypical}}$ = \$500.00, $\text{Median}_{\text{control}}$ = \$400.00, $z = 1.95$, $p = .051$), and that median actual expenses did not differ between the two conditions ($\text{Median}_{\text{atypical}}$ = \$493.00, $\text{Median}_{\text{control}}$ = \$409.00, $z = 1.17$, $p = .24$).

Figure W1

Bias Scores (Predicted – Actual Expenses) for each Week of Study 1

Bottom whisker = 1st percentile. Bottom of the box = 25th percentile. Midline = median. Top of the box = 75th percentile. Top whisker = 1.5x the height of the box. Circles = data points 1.5x–3x the height of the box. Stars = data points > 3x the height of the box.

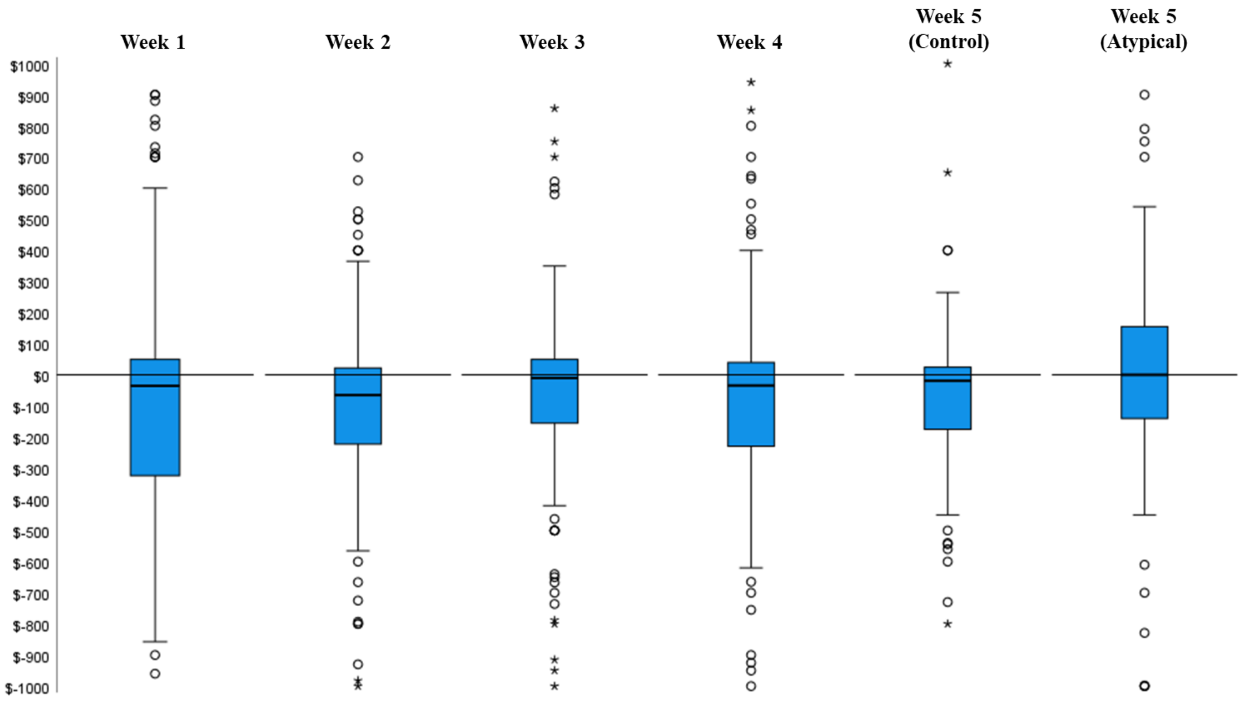

Table W3

Winsorized Mean Bias Scores and Raw Median Bias Scores for each Week of Study 1

	Week 1	Week 2	Week 3	Week 4	Week 5 (Control)	Week 5 (Atypical)
Mean Bias Score	-$216.88**	-$171.25**	-$143.61**	-$154.93**	-$78.92*	$7.07
Median Bias Score	-$35.00**	-$65.00**	-$10.00**	-$34.00**	-$19.00**	$0.00
SD	698.69	474.07	474.45	449.58	323.39	426.80

Notes: $*p < .05$, $**p < .01$. Mean bias scores and SD's were calculated after winsorizing the distribution of bias scores at the 5th and 95th percentile. Winsorized mean bias scores were compared against zero using a parametric one-sample t-test. Raw median bias scores were compared against zero using a non-parametric one-sample Wilcoxon signed ranks test.

Monthly Expense Prediction Bias. Both the winsorized mean bias score (Mean = -$751.77, SD = 1675.62) and raw median bias score (Median = -$350.00) were significantly different from zero, indicating that consumers under-predicted their monthly expenses

(parametric one-sample t-test for winsorized mean: $t(186) = -6.14$, $p < .001$; non-parametric one-sample Wilcoxon signed rank test for raw median: $z = -5.10$, $p < .001$).

Perceived Typicality. To test our hypothesis that people predict their expenses will be more typical in the future than in the past (H1a), we compared reported vs. predicted expense typicality at T1, T2, T3, and T4 using parametric paired samples t-tests with no participants excluded from the analysis. In other words, we tested whether or not participants predicted their expenses would be more typical in week 2 than week 1, week 3 than week 2, and so on. As illustrated in Figure W2 and Table W4, participants predicted their expenses would be more typical in the next (vs. past) week at all four points in time. Figure W2 also illustrates that our atypical intervention reversed this tendency at T4.

To test our hypothesis that perceived typicality of future expenses is negatively correlated with expense predictions (H1b), we analyzed the non-parametric correlation (Spearman's rho) between perceived typicality of future expenses and weekly expense predictions for each week of the study, as well as for the month. Perceived typicality of future expenses was negatively correlated with weekly expense predictions at T0 ($r(185) = -.32$, $p < .001$), T2 ($r(185) = -.23$, $p = .002$), and T4 ($r(185) = -.22$, $p = .003$), as well as with monthly predictions ($r(185) = -.15$, $p = .037$). The correlations at T1 and T3 were directionally consistent, though not significant (T1: $r(185) = -.10$, $p = .17$; T3: $r(185) = -.04$, $p = .64$).

Figure W2

Mean Reported Expense Typicality for the Past Week vs. Mean Predicted Expense Typicality for the Next Week for each Week of Study 1

Error Bars Represent 95% Confidence Intervals

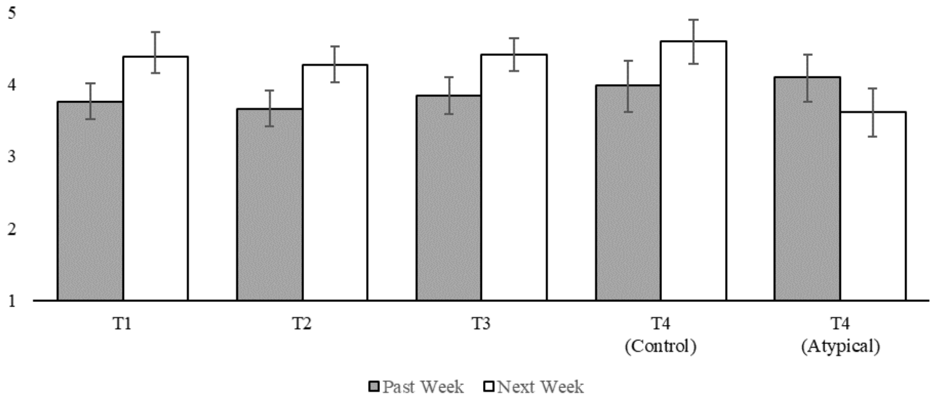

Table W4

Paired Samples T-tests Comparing Perceived Typicality of Reported Expenses for the Past Week vs. Perceived Typicality of Predicted Expenses for the Next Week in Study 1

	T1	T2	T3	T4 (Control)	T4 (Atypical)
t-stat	3.79	4.37	3.74	2.98	2.27
p -value	<0.001	< 0.001	<0.001	0.004	0.025

STUDY 2

Replicating Study 1. The results observed in Study 1 were directly replicated in the control condition of Study 2. Supporting H1a, participants in the control condition of Study 2 predicted their expenses for the next week would be significantly more typical than their expenses for the past week ($M_{pastweek}$ = 4.39, $SD_{pastweek}$ = 1.77, $M_{nextweek}$ = 4.62, $SD_{nextweek}$ = 1.69; paired-samples t-test: t(429) = -3.21, *p* = .001, d = .16). Supporting H1b, the non-parametric correlation (Spearman's rho) between perceived typicality of future expenses and expense

predictions was significant and negative (rho(428) = -.19, $p < .001$). Finally, as detailed below, participants in the control condition predicted lower expenses for the next week as compared to the past week. We next expand our analysis to test for differences in perceived typicality and expenses across all three conditions.

Expense Prediction Bias (Predicted – Recalled Expenses). Table W5 summarizes the non-parametric median analysis performed on the Study 2 expense data. First, by reading the control and typical columns vertically, it can be seen that the median of predicted expenses in these conditions is significantly lower than the median of recalled expenses. Second, by reading the atypical column vertically it can be seen that the median of predicted expenses in this condition is significantly *higher* than the median of recalled expenses. This provides support for H2 because people tend to remember the past in slightly optimistic terms (Buehler, Griffin, and Peetz 2010), which suggests that "over-prediction" in the context of prediction vs. recall translates into more accurate predictions in the context of prediction vs. reality (i.e., the expenses a consumer actually incurs during the target week). Finally, by reading horizontally across the top two rows of the table, it can be seen that the median of recalled expenses did not differ by condition, and that the median of predicted expenses did. Contrast analysis confirms that the median of predicted expenses did not differ between the control and typical conditions ($p = .58$), and that the median of predicted expenses was significantly higher in the atypical condition than in the control and typical conditions (p's $< .001$).

Table W5

Non-Parametric Median Tests for Recalled and Predicted Expenses in Study 2

Wilcoxon Signed Ranks: Tests for median differences between recalled and predicted expenses within condition (i.e., within each column). Kruskal-Wallis H: Tests for median differences across conditions (i.e., across each row).

	Control	Typical	Atypical	Kruskal-Wallis H Test
**Median Recalled Expenses**	$250.00	$220.00	$250.00	p = .42
**Median Predicted Expenses**	$200.00	$200.00	$300.00	p < .001
**Wilcoxon Signed Ranks Tests**	p = .017	p = .016	p = .003	

Table W6 summarizes the parametric winsorized mean analysis performed on the Study 2 expense data. First, by reading the control and typical columns vertically, it can be seen that the winsorized mean bias score in these conditions is significantly less than zero, indicating that participants in these conditions predicted lower expenses for the next week than they recalled for the past week. Second, by reading the atypical column vertically it can be seen that the winsorized mean bias score in the atypical condition is significantly greater than zero. So, as above, this analysis suggests that our atypical intervention not only neutralized the tendency to underpredict in this study, it reversed it. Finally, by reading horizontally across the bottom two rows of the table, it can be seen that the winsorized mean bias scores in the control and typical conditions did not significantly differ, and that the winsorized mean bias score in the atypical condition was significantly higher than in the control and typical conditions. (NB: A one-way ANOVA with condition as the IV and winsorized bias scores as the DV produced a significant omnibus test ($F(2, 1088) = 18.38$, $p < .001$).

Table W6

Winsorized Mean EPB Scores in each Condition of Study 2

	**Control** (1)	**Typical** (2)	**Atypical** (3)
**Mean EPB** (SD)	-$31.27 (246.61)	-$34.08 (176.53)	$92.83 (463.40)
**t-test** (diff from zero)	*t* (430) = -2.63 *p* = .009	*t* (340) = -3.57 *p* < .001	*t* (318) = -3.58 *p* < .001
**Contrast** (1 vs. 2)	t(1088) = .13 *p* = .90		
**Contrast** (1 & 2 vs. 3)	t(1088) = 6.06 *p* < .001		

Note: The distribution of EPB scores was winsorized at the 5th and 95th percentile of each condition. This presents the resultant means and standard deviations with which statistical tests were performed.

Atypical Expense Listing Task. A one-way ANOVA with condition (control vs. typical vs. atypical) as the IV and number of atypical expenses (i.e., expenses predicted to occur in the next week that didn't occur in the past week) as the DV revealed a significant effect of condition (F(2, 1105) = 6.88, *p* = .001). Planned contrasts further revealed that the number of expenses listed in the atypical condition ($M_{atypical}$ = 1.63, $SD_{atypical}$ = 1.62) was significantly higher than in the control and typical conditions ($M_{control}$ = 1.24, $SD_{control}$ = 1.47, $M_{typical}$ = 1.39, $SD_{typical}$ = 1.60; t(1088) = 3.03, *p* = .003), and that the number of expenses listed in the control and typical conditions did not differ significantly (t(1088) = 1.32, *p* = .19). Furthermore, the non-parametric correlation between the number of expenses listed and predictions is positive and significant in all three conditions (*r*'s > .16, *p*'s < .01). Finally, a one-way ANOVA with condition as the IV and average dollar amount of atypical expenses as the DV reveals no effect of condition (F(2, 565) = .84, *p* = .44).

Perceived Typicality of Future Expenses. A one-way ANOVA with intervention condition (control vs. typical vs. atypical) as the independent variable and perceived typicality of future expenses as the dependent variable revealed a significant effect of condition on perceived typicality of future expenses (F(2, 1086) = 35.16, $p < .001$). Planned contrasts further revealed that perceived typicality was virtually identical in the control and typical conditions ($M_{control}$ = 4.62, $SD_{control}$ = 1.69, $M_{typical}$ = 4.63, $SD_{typical}$ = 1.62, $t(1086) = -.10$, $p = .92$), but significantly lower in the atypical condition ($M_{atypical}$ = 3.70, $SD_{atypical}$ = 1.65, $t(1086) = 8.38$, $p < .001$).

In sum, the results of the robustness tests for Studies 1 and 2 are highly consistent with the results presented in the main text.

WEB APPENDIX D:

DETAILS OF THE DATA TRANSFORMATION PROCESS IN STUDIES 1 AND 2

Table W7
Details of the Data Transformation Process for Week 5 of Study 1

Study 1 Distributional Analysis for Week 5 Data

Expense distributions w/ no exclusions and no transformation (n = 187)

	Control Condition (n = 94)					Atypical Condition (n = 93)					Levene's Test of HOV	
	Median	Mean	SD	Skew	Normality Test p-value	Median	Mean	SD	Skew	Normality Test p-value	Levene Statistic	p-value
**Reported**	$409.00	$722.15	960.29	4.17	< .001	$493.00	$995.82	1910.35	5.61	< .001	2.79	0.097
**Predicted**	$400.00	$620.88	710.94	2.20	< .001	$500.00	$867.88	1326.50	5.94	< .001	2.20	0.139

Expense distributions after excluding outliers (n = 183)

	Control Condition (n = 92)					Atypical Condition (n = 91)					Levene's Test of HOV	
	Median	Mean	SD	Skew	Normality Test p-value	Median	Mean	SD	Skew	Normality Test p-value	Levene Statistic	p-value
**Reported**	$431.50	$733.22	967.57	4.14	< .001	$493.00	$852.70	1232.91	5.05	< .001	0.47	0.495
**Predicted**	$400.00	$572.97	630.42	2.24	< .001	$500.00	$870.47	1336.37	5.94	< .001	3.83	0.052

Expense distributions after excluding outliers and applying LN transformation (n = 183)

	Control Condition (n = 92)					Atypical Condition (n = 91)					Levene's Test of HOV	
	Median*	Mean*	SD	Skew	Normality Test p-value	Median*	Mean*	SD	Skew	Normality Test p-value	Levene Statistic	p-value
**Reported**	6.0668 ($431.30)	6.0992 ($445.50)	0.98	0.16	0.964	6.2005 ($493.00)	6.2005 ($493.00)	1.08	-0.35	0.149	0.06	0.810
**Predicted**	5.9915 ($400.01)	5.9013 ($365.51)	0.94	0.27	0.147	6.2146 ($500.00)	6.2139 ($500.00)	1.06	-0.05	0.793	1.03	0.310

*Values in parentheses represent exponentiated medians and means.

Table W8
Details of the Data Transformation Process for Study 2

Study 2 Distributional Analysis

Expense distributions w/ no exclusions and no transformation (n = 1108)

	Control Condition (n = 437)					Typical Condition (n = 347)					Atypical Condition (n = 324)					Levene's Test of HOV	
	Median	Mean	SD	Skew	Normality Test p-value	Median	Mean	SD	Skew	Normality Test p-value	Median	Mean	SD	Skew	Normality Test p-value	Levene Statistic	p-value
**Recalled**	$250.00	$451.60	885.06	6.81	< .001	$220.00	$365.00	426.21	2.79	< .001	$250.00	$457.75	698.74	5.14	< .001	3.82	0.022
**Predicted**	$200.00	$388.94	568.57	3.80	< .001	$200.00	$356.16	606.70	6.99	< .001	$300.00	$584.34	938.99	3.87	< .001	17.72	< .001

Expense distributions after excluding outliers (n = 1048)

	Control Condition (n = 416)					Typical Condition (n = 332)					Atypical Condition (n = 300)					Levene's Test of HOV	
	Median	Mean	SD	Skew	Normality Test p-value	Median	Mean	SD	Skew	Normality Test p-value	Median	Mean	SD	Skew	Normality Test p-value	Levene Statistic	p-value
**Recalled**	$247.50	$426.33	793.74	7.65	< .001	$200.00	$342.99	382.21	2.92	< .001	$250.00	$459.93	701.04	5.25	< .001	4.93	0.007
**Predicted**	$200.00	$380.04	538.01	3.82	< .001	$200.00	$336.67	516.33	7.59	< .001	$292.50	$492.03	651.27	3.04	< .001	8.14	< .001

Expense distributions after excluding outliers and applying LN transformation (n = 1048)

	Control Condition (n = 416)					Typical Condition (n = 332)					Atypical Condition (n = 300)					Levene's Test of HOV	
	Median*	Mean*	SD	Skew	Normality Test p-value	Median*	Mean*	SD	Skew	Normality Test p-value	Median*	Mean*	SD	Skew	Normality Test p-value	Levene Statistic	p-value
**Recalled**	5.5114	5.4671	1.03	0.14	0.002	5.2983	5.3633	1.01	-0.20	0.038	5.5288	5.5288	1.09	-0.02	0.278	1.09	0.340
	($247.50)	($236.77)				($200.00)	($213.43)				($251.84)	($251.84)					
**Predicted**	5.2983	5.3728	1.04	0.22	0.007	5.2983	5.2973	1.01	-0.01	0.150	5.6781	5.6027	1.11	-0.03	0.160	1.59	0.204
	($200.00)	($215.47)				($200.00)	($199.80)				($292.39)	($271.16)					

*Values in parentheses represent exponentiated medians and means.

WEB APPENDIX E:

ANALYSIS OF EXPLORATORY VARIABLES IN STUDY 2

After participants completed the Study 2 measures detailed in the main text, we had them complete five measures designed to let us to explore the relationship between EPB, financial slack (Zauberman and Lynch, 2005; Berman et al. 2016), various measures of spending, and available resources. These measures and the results they yielded are detailed below.

Financial Slack: The financial slack measure was “Using the scale below, please indicate how much spare money you expect to have in the next week, compared to an average week in your life (1 = Very little spare money; 7 = A lot of spare money).” We hypothesized that predicted slack would be higher in the control and typical conditions (vs. the atypical condition) because EPB was higher in these conditions. To test this hypothesis we performed a 3(condition: control vs. typical vs. atypical) x 2(order: predict then recall vs. recall then predict) ANOVA with slack as the dependent variable. Neither the main effects nor the interaction were significant (p’s > .24).

WTP for Dinner: This measure asked “Imagine that a friend invites you to go out for a fancy dinner next week. You will each pay for your own food and drinks. How much money would you be willing to spend on dinner, including all your food, drinks, taxes, and tip? (1 = $0-$10; 11 = More than $100).” We hypothesized that WTP would be higher in the control and typical conditions (vs. the atypical condition) because EPB was higher in these conditions. To test this hypothesis we performed a 3(condition: control vs. typical vs. atypical) x 2(order: predict then recall vs. recall then predict) ANOVA with WTP as the dependent variable. Neither the main effects nor the interaction were significant (p’s > .24).

WTP for an Emergency Loan: This measure asked "Imagine that next week you find yourself with an unexpected bill. For example, suppose that you use your vehicle to get to work, and it requires an expensive repair that is not covered by insurance. Until you get your car repaired, you will have to walk to work, which will add an extra 60 minutes onto your commute each way. Now imagine that to help cover the cost of fixing your vehicle you are able to take out a $350 loan which will need to be repaid in 2 weeks along with the lender's fee. Using the scale below, please indicate the highest lender's fee you would be willing to pay to be able to borrow the $350 (1 = $0; 11 = More than $50)." We hypothesized that WTP would be higher in the control and typical conditions (vs. the atypical condition) because EPB was higher in these conditions. To test this hypothesis we performed a 3(condition: control vs. typical vs. atypical) x 2(order: predict then recall vs. recall then predict) ANOVA with WTP as the dependent variable. Neither the main effects nor the interaction were significant (p's > .20).

Loan Repayment Confidence: This measure asked "Assuming that you took the loan offered in the previous question, how confident are you that you would be able to pay back the loan (including the lender's fee) within 2 weeks? (1 = Extremely confident; 7 = Extremely unconfident)." We hypothesized that confidence would be higher in the control and typical conditions (vs. the atypical condition) because EPB was higher in these conditions. To test this hypothesis we performed a 3(condition: control vs. typical vs. atypical) x 2(order: predict then recall vs. recall then predict) ANOVA with confidence as the dependent variable. Neither the main effects nor the interaction were significant (p's > .26).

$1,000 Allocation Task: This measure asked "Imagine that you have just inherited $1,000 that you weren't expecting. How much of the $1,000 would you use for each of the following? (Please note that your total must equal $1,000)." We hypothesized that less money would be

allocated to saving in the control and typical conditions (vs. the atypical condition) because EPB was higher in these conditions. To test this hypothesis we performed a 3(condition: control vs. typical vs. atypical) x 2(order: predict then recall vs. recall then predict) ANOVA with the sum allocated to saving as the dependent variable. The hypothesized main effect of condition did not reach significance ($p = .27$).

Available Resources: This measure asked "Imagine that you have to pay an unexpected bill immediately. For example, suppose that you require an expensive medical procedure that is not covered by insurance. Considering all possible resources available to you (including savings, borrowing, etc.), what is the maximum dollar amount that you could come up with on short notice? Please enter the amount below." This measure was included so that we could gain some insight as to whether or not EPB is associated with socio-economic status. The correlation between EPB and available resources was statistically significant but substantively weak ($r(983) = -.07$, $p = .022$).

WEB APPENDIX F:

DETAILS OF SUPPLEMENTAL STUDY A

The primary goal of this study was to further test our hypothesis that the atypical intervention increases predictions by making atypical expenses cognitively easier to retrieve (H2). To accomplish this, we ran a think aloud experiment that allows us to examine the effect of the intervention (versus control) on both prediction content and structure, where content is defined as the different types of thoughts that come to mind during prediction, and structure is defined as the order in which these thoughts come to mind. A secondary goal of this study was to further explore a motivational account of the expense prediction bias, and examine the potential relationship between need-for-cognition and predictions.

Method

Participants and Procedure. Four hundred and one participants were recruited for this preregistered study (https://aspredicted.org/ni68b.pdf) about consumer decision making through Amazon Mechanical Turk (31.2% female, M_{age} = 34.6). We randomly assigned participants to predict their expenses for the next week in one of three conditions: control, typical, or atypical. The prediction instructions in each condition were the same as in Study 2, but in this study we also asked participants to complete the following think aloud task as they made their prediction: "Please type every thought that enters your mind as you think about the following question and decide on your answer. How much do you estimate your total expenses will be for the next week (i.e., the next 7 days)?" So, for example, participants in the atypical condition listed three reasons why their expenses for the next week might be different from a typical week, then completed the think aloud task. We used a written think aloud task in this study rather than the verbal task used in our pilot study because past research has shown these methods to be equally valid (Buehler et

al. 2010), and the written task does not require online participants to have a microphone. After completing the think aloud task participants were asked to "Please enter your estimated total expenses (in dollars) for the next week." Participants then completed the short-form Need for Cognition (NFC) scale (Cacioppo and Petty 1984) so we could explore whether it is associated with expense predictions. NFC was not correlated with predictions in any of the three experimental conditions (*r*'s < .09, *p*'s > .39) nor did it moderate the effect of our intervention on predictions versus control (b = -.08, 95% CI = [-.28, .12], *t*(181) = -.76, *p* = .45), so it is not discussed further.

As with the think aloud pilot study, we had two research assistants who were blind to experimental condition code the content of the think aloud data for whether or not each participant referenced typical spending, future-oriented spending (i.e., atypical spending specific to the next week), and/or an adjustment for unexpected spending. We also had them code whether or not each participant referenced the motivation to spend less or save more so that we could explore the extent to which this type of motivated cognition might be driving the bias. Finally, we had the research assistants code the structure of the data by recording which type of thought each participant listed first.

As per our preregistration, expense predictions were LN-transformed, and data was excluded from participants who provided gibberish responses to the think aloud question (e.g., by copying and pasting the question into the answer box), reported technical difficulties, and/or straight-lined through the NFC scale. After exclusions, our effective sample size for this study was 271 participants (35.8% female, M_{age} = 35.8).

Results

Prediction Amount. Supporting H2, planned contrasts revealed that predictions in the atypical intervention condition (M = \$393.39, 95% CI = [295.78, 523.22]) were 41.9% higher than in the control condition (M = \$277.27, 95% CI = [213.79, 359.60]; $t(268) = 1.90$, $p = .058$), and 67.1% higher than in the typical condition (M = \$235.36, 95% CI = [187.45, 295.48]; $t(268) = 2.67$, $p = .008$). Furthermore, consistent with our proposition that expense predictions are naturally based on typical expenses, predictions in the control and typical conditions did not differ significantly ($t(268) = .91$, $p = .37$).

Prediction Content. Table W9 summarizes the results of our prediction content analysis. Supporting our proposition that the atypical intervention increases predictions by making atypical expenses more accessible, a higher proportion of participants referenced future-oriented spending in the atypical condition than in the control condition (mean difference = 16.5%, 95% CI = [.29%, 31.91%], $\chi_{(1)} = 3.94$, $p = .047$) or typical condition (mean difference = 27.3%, 95% CI = [10.31%, 42.35%], $\chi_{(1)} = 9.84$, $p = .002$). The proportion of participants in the control and typical conditions who referenced future-oriented spending did not differ significantly (mean difference = 10.8%, 95% CI = [-3.90%, 24.18%], $\chi_{(1)} = 2.10$, $p = .15$). The proportion of participants who referenced typical spending, an adjustment for unexpected spending, and a motivation to save money or minimize spending did not differ significantly across the three conditions. Notably, very few participants made a motivational reference.

Table W9
Proportion of Participants in Each Condition of Supplemental Study A Whose Prediction Included Each Type of Thought Classification

Thought Classification	Control Condition	Typical Condition	Atypical Condition	Examples
Typical Spending	91.8%[a]	85.5%[a]	84.5%[a]	"I am thinking about what my typical expenses are, such as food, bills, transportation."
Future-Oriented Spending	31.8%[a]	21.0%[a]	48.3%[b]	"There was a pajama set at Target I really liked that I hope is still there for me to purchase."
Adjustment for Unexpected Spending	11.8%[a]	14.5%[a]	19.0%[a]	"I also have to account for a few unexpected expenses and impulse buys that occasionally come up."
Motivation to Save Money or Minimize Spendin[illegible]	4.7%[a]	3.2%[a]	3.4%[a]	"I want to save as much money as I can."

[a]Indicates that a proportion does not differ significantly from the other proportions in the same row (p > .05).
[b]Indicates that a proportion differs signficantly from the next highest proportion in the same row (p < .05)

Prediction Structure. Table W10 summarizes the results of our prediction structure analysis. Supporting our proposition that the atypical intervention increases predictions by making atypical expenses more accessible, a higher proportion of participants listed future-oriented spending as their first thought in the atypical condition than in the control condition (mean difference = 15.7%, 95% CI = [1.85%, 29.86%], $\chi_{(1)}$ = 4.97, p = .026) or typical condition (mean difference = 21.3%, 95% CI = [6.95%, 35.08%], $\chi_{(1)}$ = 8.43, p = .004). A higher proportion of participants in the atypical condition also referenced an adjustment for unexpected expenses as their first thought, as compared to the control condition (mean difference = 7.4% 95% CI = [.27%, 17.47%], $\chi_{(1)}$ = 4.66, p = .031). This pattern flips for references to typical spending: a higher proportion of participants in the control condition referenced typical spending first, as compared to the atypical condition (mean difference = 23.6% 95% CI = [8.03%, 38.29%], $\chi_{(1)}$ = 8.95, p = .003), and this was also true in the typical condition (mean difference = 27.1% 95% CI = [10.57%, 41.88%], $\chi_{(1)}$ = 10.24, p = .001). Notably, very few participants referenced a motivational thought first.

Table W10
The Proportion of Participants in Each Condition of Supplemental Study A Who Referenced Each Type of Thought Classification First

Thought Classification	Control Condition	Typical Condition	Atypical Condition
Typical Spending	78.8%[a]	82.3%[a]	55.2%[b]
Future-Oriented Spending	15.3%[a]	9.7%[a]	31.0%[b]
Adjustment for Unexpected Spending	1.2%[a]	3.2%[a]	8.6%[c]
Motivation to Save	1.2%[a]	1.6%[a]	1.7%[a]

[a]Indicates that a proportion does not differ significantly from the other proportions in the same row (p > .05).
[b]Indicates that a proportion differs signficantly from the next highest proportion in the same row (p < .05)

Discussion

Supplemental Study A complements and extends Studies 1 and 2 by providing further evidence that consumers' expense predictions are based on typical expenses, and that increasing the accessibility of atypical expenses increases predictions (H2). This study also provides evidence that expense predictions are not consciously influenced by motivation to save, and that expense predictions are not associated with Need for Cognition. This latter result suggests that the desire to think deeply is not enough to increase expense predictions; it is the content and structure of one's thoughts that matter.

WEB APPENDIX G:

NON-PARAMETRIC ANALYSIS FOR STUDY 3

Figure W3 plots the median expense prediction in each condition. Listing three reasons why future expenses might be different than usual increased the median prediction by 42.9% vs. listing zero reasons ($p < .001$), and listing ten reasons increased the median prediction by 71.4% vs. listing zero reasons ($p < .001$). Listing ten reasons also increased prediction by 20.0% vs. listing three reasons, but this contrast did not reach significance ($p = .46$).

Figure W3
Median Expense Prediction in Each Condition of Study 3

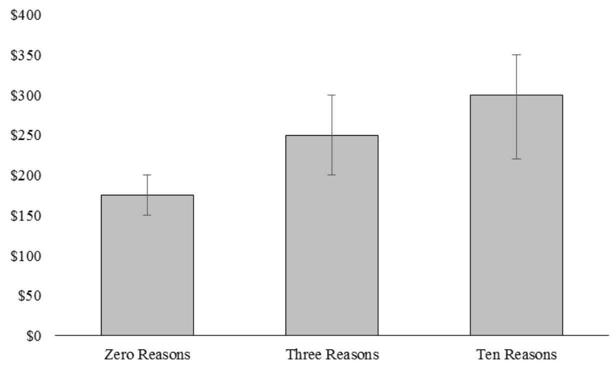

WEB APPENDIX H:

RESULTS OF SUPPLEMENTAL STUDY B

Method

Participants and Procedure. Four hundred participants completed this preregistered study (https://aspredicted.org/u6tm8.pdf) on Prolific Academic (47.5% female, M_{age} = 34.4). This study was the same as the original in every way, except the underlying distributions consisted of $5 intervals rather than $10 intervals. For convenience, we've included the distributions from the original study and the replication study below.

Distributions from the Original Study

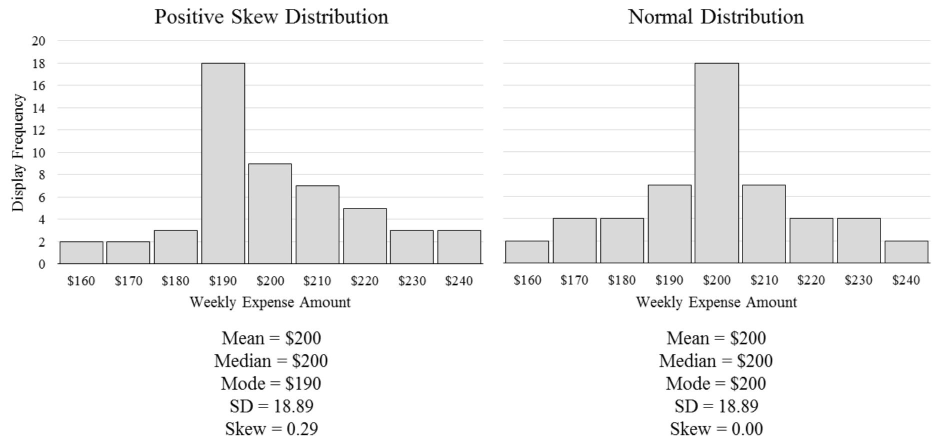

Distributions from the Replication Study

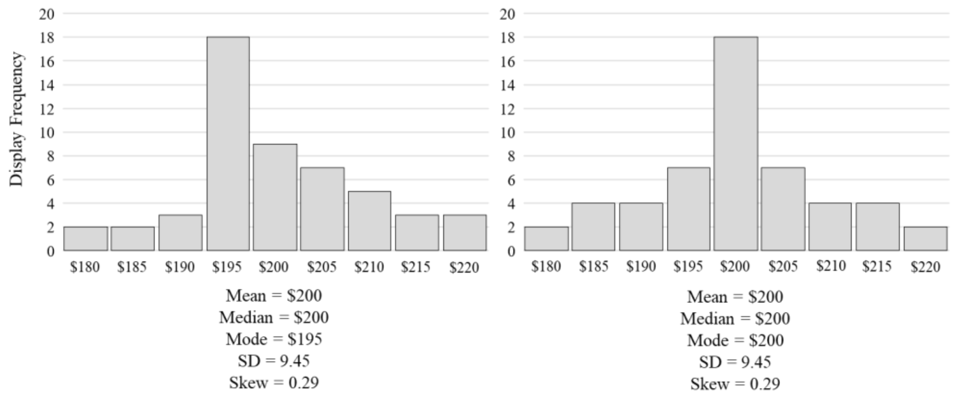

Results

Expense Predictions. On average, participants in both conditions made predictions at the mode of the distribution they had seen, which led participants in the positive skew condition to make lower predictions than participants in the normal distribution condition. Expense predictions were significantly lower in the positive skew condition (M = \$194.57, SD = 22.55) than in the normal distribution condition (M = \$200.16, SD = 7.12), as revealed by an independent samples t-test ($t(398) = 3.35$, $p = .001$ $d = .34$). A pair of one-sample t-tests further revealed that expense predictions in the positive skew condition were significantly lower than the \$200 mean of the underlying distribution ($t(198) = -3.40$, $p < .001$), but not significantly different than the \$195 mode ($t(198) = -0.27$, $p = .79$). A one-sample t-test also showed that predictions in the normal distribution condition did not differ significantly from \$200 ($t(200) = 0.33$, $p = .74$). As in the original study, we also found that the proportion of participants who predicted spending *less* than \$200 was significantly higher in the positive skew condition than in the normal distribution condition (33.2% in the skewed condition vs. 13.9% in the normal condition; $\chi_{(1)} = 20.67$, $p < .001$). Moreover, the proportion of participants who predicted spending *equal* to \$200 was significantly lower in the skewed condition than in the normal condition (50.3% in the skewed condition vs. 70.1% in the normal condition; $\chi_{(1)} = 16.33$, $p < .001$), but the proportion of participants who predicted spending *more* than \$200 was very similar in both conditions (16.6% in the skewed condition vs. 16.9% in the normal condition; $\chi_{(1)} = .01$, $p = .94$).

WEB APPENDIX I:

STUDY 5 ROBUSTNESS TESTS

ANOVA Results with Control Variables

This section details the results of the 2(condition: control vs. atypical) × 2(category: online vs. grocery) × 2(expenses: predicted vs. actual) mixed model ANOVA presented in the main text when mean, standard deviation, and range of past spending are included in the model as control variables. Each of these control variables displays strong positive skew, so they were normalized for this analysis using a LN-transformation.

Figure W4 presents the results of the ANOVA when the control variables are included in the model. There was a significant main effect of category ($F(1, 1731) = 61.12$, $p < .001$, $\eta_p^2 = .03$), a significant main effect of expenses ($F(1, 1731) = 23.69$, $p < .001$, $\eta_p^2 = .01$), and a significant main effect of condition ($F(1, 1731) = 6.12$, $p = .014$, $\eta_p^2 = .01$). There was also a significant two-way interaction between expenses and condition ($F(1, 1731) = 10.14$, $p = .001$, $\eta_p^2 = .01$), and a significant three-way interaction between expenses, condition, and category ($F(1, 1731) = 7.74$, $p = .005$, $\eta_p^2 = .004$). Replicating the original analysis, online expense predictions were 39.5% higher in the atypical condition (*M* = £142.59, 95% CI = [126.22, 161.26]) than in the control condition (*M* = £102.21, 95% CI = [90.74, 115.12]; $F(1, 926) = 14.63$, $p < .001$), but actual online expenses in the atypical condition (*M* = £139.77, 95% CI = [124.96, 156.34]) did not differ significantly from actual online expenses in the control (*M* = £150.66, 95% CI = [135.10, 168.01]; $F(1, 926) = .87$, $p = .35$). Also replicating the main analysis, predicted grocery expenses were only directionally higher in the atypical condition than in the control ($M_{atypical}$ = £81.70, 95% CI = [76.55, 87.18]; $M_{control}$ = £75.94, 95% CI = [71.38,

80.88]; *F*(1, 802) = 2.48, *p* = .12), and actual grocery expenses in each condition did not differ significantly ($M_{atypical}$ = £99.58, 95% CI = [91.38, 108.42]; $M_{control}$ = £95.20, 95% CI = [87.71, 103.34]; *F*(1, 802) = .55, *p* = .46).

Figure W4
Predicted vs. Actual Expenses in Each Condition of Study 5 When Controlling for Mean, Median, Standard Deviation, and Range of Past Expenses

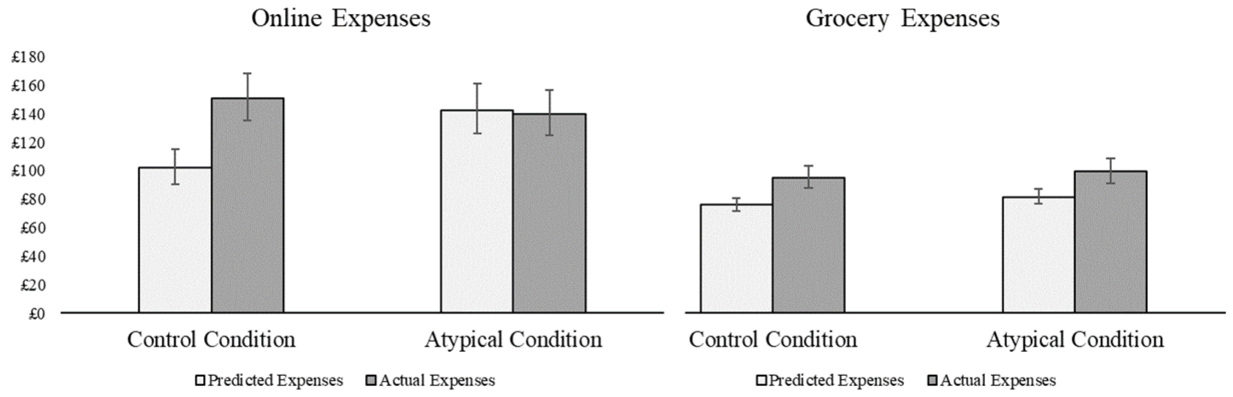

Non-Parametric Median Analysis

Consistent with the robustness tests we performed for Studies 1 and 2, this analysis was performed with the raw, untransformed data.

Online Expenses. The median bias score in the intervention condition ($3.14) was significantly lower than the median bias score in the control condition (-$26.74), as revealed by a independent-samples non-parametric median test (*t*-stat = 5.11, *p* = .028). The median prediction in the intervention condition ($150.00) was significantly higher than the median prediction in the control condition ($100.00; *t*-stat = 10.86, *p* = .001), but the median of actual online expenses

incurred during the week of the experiment did not differ between the two conditions (*Intervention* = \$154.95, *Control* = \$159.13, *t*-stat = .05, *p* = .87).

Grocery Expenses. The median bias score in the intervention condition (\$29.98) did not differ significantly from the median bias score in the control condition (\$38.42), as revealed by an independent-samples non-parametric median test (*t*-stat = 1.70, *p* = .22). The median prediction in the intervention condition (\$100.00) was marginally higher than the median prediction in the control condition (\$80.00; *t*-stat = 3.48, *p* = .073), but the median of actual online expenses incurred during the week of the experiment did not differ between the two conditions (*Intervention* = \$114.27, *Control* = \$123.80, *t*-stat = .28, *p* = .65).

In sum, the results of this non-parametric median analysis are wholly consistent with the results of the parametric mean analysis reported in the main text.

Winsorized Mean Analysis

Consistent with the robustness tests for Studies 1 and 2, we winsorized participants' raw, untransformed bias scores (calculated as predicted minus actual expenses) at the 5th and 95th percentile of each condition, then performed parametric t-tests to compare the size of the bias in each condition. The winsorized mean bias score for participants in the online expenses control condition (*M* = -\$114.04, *SD* = 351.09) was significantly worse than the winsorized mean bias score for participants in the online expenses intervention condition (*M* = -\$55.23, *SD* = 300.36), as revealed by an independent samples t-test (*t*(929) = 2.74, *p* =.006). The winsorized mean bias score for participants in the grocery expenses intervention condition (*M* = -\$56.21, *SD* = 110.71) was somewhat better than the winsorized mean bias score for participants in the grocery expenses control condition (*M* = -\$72.32, *SD* = 121.31; *t*(805) = 1.97, *p* = .050), which is consistent with our finding that the intervention influence grocery expense predictions to some

degree. In sum, the results of this winsorized mean analysis are consistent with the results of the parametric mean analysis reported in the main text.

WEB APPENDIX J:

RESULTS OF SUPPLEMENTAL STUDY C

The primary goal of this study was to more cleanly determine if perceived typicality of future expenses differs between weekly and monthly predictions. This is important from a theoretical perspective because it is possible that typical expenses do not dominate predictions for longer periods of time (e.g., a month) like they do for shorter periods of time (e.g., a week). For example, a more distant prediction horizon may be associated with more uncertainty, which could cause consumers to build an error term into their prediction that accounts for atypical expenses (Ülkümen et al. 2008). If true, we would expect to observe lower perceived typicality for monthly (vs. weekly) predictions in the absence of an intervention. It would also be reasonable to expect a weaker effect of the atypical intervention on perceived typicality for monthly (vs. weekly) predictions, because if monthly predictions are less influenced by typical expenses there should be less room for perceived typicality to be shifted downward by the intervention. This study tests these possibilities, and examines if the atypical intervention is capable of increasing monthly as well as weekly expense predictions. This is a matter of practical importance given that Study 1 demonstrates that an expense prediction bias exists for both time periods.

Method

Participants and Procedure. We recruited 601 participants (48.6% female, M_{age} = 37.93) from Amazon Mechanical Turk to take part in a consumer expense survey. Participants were randomly assigned to predict their expenses in a 2 (prediction time frame: week vs. month) × 2 (intervention condition: control vs. atypical) between-subjects design that utilized the same

prediction prompts as in study 1, modified where necessary for monthly predictions. Participants also reported perceived typicality and prediction confidence as in Study 1. As per our preregistration (https://aspredicted.org/c8qg9.pdf), expense predictions were winsorized at the 5th and 95th percentile of each condition and LN-transformed.

Results

Weekly vs. Monthly Prediction Typicality. Perceived typicality did not differ as a function of time period, only as a function of intervention condition. Specifically, a 2 (prediction time period: week vs. month) × 2 (intervention condition: control vs. atypical) ANOVA with perceived typicality as the dependent variable revealed a main effect of intervention condition such that perceived typicality was higher in the control condition (*M* = 5.20, SD = 1.55) than in the atypical condition (*M* = 3.11, SD = 1.59; $F(1, 597) = 263.73$, $p < .001$, $\eta_p^2 = .31$). However, there was no main effect of time period ($F(1, 597) = .72$, $p = .40$) and no intervention condition by time period interaction ($F(1, 597) = 2.09$, $p = .15$).

Monthly Expense Predictions. The impact of the atypical intervention on monthly expense predictions was consistent with its impact on weekly expense predictions in the final week of Study 1: Monthly expense predictions were 24.6% higher in the atypical condition (*M* = $1505.98, 95% CI = [$1314.88, $1724.86]) than in the control condition (*M* = $1208.34, 95% CI = [$1049.32, $1391.59]), as revealed by an independent-samples t-test ($t(297) = 2.22$, $p = .027$). Furthermore, perceived typicality was found to be significantly lower in the atypical condition (*M* = 2.97, 95% CI = [2.74, 3.21]) than in the control condition (*M* = 5.24, 95% CI = [4.99, 5.50]; $t(297) = 12.91$, $p < .001$). Prediction confidence did differ between the two conditions ($M_{atypical} = 4.79$, 95% = [4.56, 5.01]; $M_{control} = 5.39$, 95% $CI_{control}$ = [5.19, 5.60]; $t(297) = 3.94$, $p <$

.001), but the focal comparison between predicted expenses in the atypical and control conditions remained significant after controlling for confidence ($F(1, 296) = 8.33$, $p = .004$).

Weekly Expense Predictions. The pattern of results observed in the final week of Study 1 was replicated: Weekly expense predictions were 61.0% higher in the atypical condition (*M* = $335.26, 95% CI = [$290.03, $387.53]) than in the control condition (*M* = $208.20, 95% CI = [$181.22, $239.20]), as revealed by an independent-samples t-test ($t(300) = 4.67$, $p < .001$). Furthermore, perceived typicality was found to be significantly lower in the atypical condition (*M* = 3.27, 95% CI = [2.98, 3.55]) than in the control condition (*M* = 5.16, 95% CI = [4.93, 5.40]; $t(300) = 10.16$, $p < .001$). Finally, it was found that prediction confidence did not differ by condition ($M_{atypical} = 5.20$, 95% = [4.99, 5.40]; $M_{control} = 5.35$, 95% $CI_{control}$ = [5.17, 5.54]; $t(300) = 1.13$, $p = .26$).

Discussion

The results of this study reveal that perceived typicality of future expenses differs only as a function of intervention condition (control vs. atypical) and not as a function of prediction time period (week vs. month). Moreover, the impact of the atypical intervention on perceived typicality is roughly equal across time periods. In tandem, these findings suggest that the influence of typical expenses on predictions—and the influence of the atypical intervention—is similar across these two common prediction time frames. The results of this study also demonstrate that prompting consumers to consider atypical expenses can increase monthly expense predictions as well as weekly expense predictions. Taken together with the results of Study 1—which showed that consumers under-predict their monthly expenses as compared to their actual expenses during the target month—this suggests that the atypical intervention is capable of reducing monthly expense prediction bias as well as its weekly cousin.

WEB APPENDIX K:

RESULTS OF SUPPLEMENTAL STUDY D

The results of Studies 1, 2, 3, 5, and Supplemental Studies A and B show that the atypical intervention increases predictions. The purpose of this study is to extend those results by examining the effect of the atypical intervention on downstream decision-making. Specifically, this study looks at whether or not the atypical intervention can increase saving intentions by increasing expense predictions.

Method

Participants and Procedure. Five hundred and ninety-five US residents were recruited through Amazon Mechanical Turk to participate in a short study about financial decision making (53.4% female, $M_{\text{age}} = 37.0$). Participants were randomly assigned to one of four conditions in a 2(Prediction Condition: Control vs. Atypical Intervention) x 2(Prediction Period: Next Week vs. Next Month) design. Participants predicted their expenses as in Supplemental Study C. To measure intention to save, participants were asked to imagine they had just received $1,000, then "How much of the $1,000 would you save to help cover your expenses for the next week/month?" We also measured the following preregistered control variable to minimize error variance in the analyses pertaining to savings: "If you think you are going to *spend* more than you typically do in the future, you should try to compensate by *saving* more than you typically do in the present." (Strongly Disagree =1, Strongly Agree = 7).[2] Our preregistered hypotheses for this study were that the atypical intervention would increase expense predictions versus control

[2] A 2(Prediction Condition: Control vs. Atypical Intervention) × 2(Prediction Period: Next Week vs. Next Month) ANOVA with this "compensation" variable as the DV revealed no significant effects (p's > .36). However, it was found to be significantly correlated with the savings intention variable ($r(590) = .15$, $p < .001$).

(H2), and that higher expense predictions would be associated with higher savings. As per our preregistration, expense predictions were LN-transformed for all analyses (https://aspredicted.org/n3vr9.pdf).

Results

Expense Predictions. A 2(Prediction Condition: Control vs. Atypical Intervention) x 2(Prediction Period: Next Week vs. Next Month) ANOVA with expense predictions as the dependent variable replicated the effect of the atypical intervention: predictions in the atypical conditions were higher than in the control conditions ($M_{atypical}$ = $596.45, 95% $CI_{atypical}$ = [522.70, 686.08], $M_{control}$ = $484.44, 95% $CI_{control}$ = [424.54, 552.80], $F(1, 592) = 4.83$, $p = .028$, $\eta_p^2 = .01$). There was also an unsurprising main effect of time period, such that monthly predictions were significantly higher than weekly predictions (M_{month} = $1378.84, 95% CI_{month} = [1,207.13, , 1,574.98], M_{week} = $210.61, 95% CI_{week} = [184.01, 241.05], $F(1, 592) = 379.07$, $p < .001$, $\eta_p^2 = .39$). There was no interaction between prediction condition and time period ($F(1, 592) = 1.61$, $p = .21$).

Savings. A 2(Prediction Condition: Control vs. Atypical Intervention) x 2(Prediction Period: Next Week vs. Next Month) ANOVA with savings as the dependent variable and spend-save compensation as a covariate revealed a main effect of prediction condition such that savings in the atypical conditions were 16.6% higher than in the control conditions ($M_{atypical}$ = $699.38, 95% $CI_{atypical}$ = [663.08, 735.69], $M_{control}$ = $599.79, 95% $CI_{control}$ = [564.69, 634.89], $F(1, 590) = 15.00$, $p < .001$, $\eta_p^2 = .03$). The ANOVA also revealed a main effect of time period such that intended savings were higher in the monthly conditions than the weekly conditions (M_{month} = $726.68, 95% CI_{month} = [691.16, 762.20], M_{week} = $572.50, 95% CI_{week} = [536.60, 608.39], F(1,

590) = 35.95, $p < .001$, $\eta_p^2 = .06$). There was no interaction between prediction condition and time period ($F(1, 590) = 1.54$, $p = .22$).

Mediation Analysis. To test the expectation that the atypical intervention increases intentions to save by increasing expense predictions we performed a mediation analysis with prediction condition as the independent variable (atypical = 1, control = 0), expense predictions as the mediator, and savings as the dependent variable. The spend-save compensation variable was again included as a control, as was prediction time period to account for the 2x2 nature of the study design. (Both of these control variables were included in our pre-registered mediation analysis plan.) The indirect effect of prediction condition on savings was significant (indirect effect = 5.28, SE = 3.90, 95% CI = [.17, 16.57]). Specifically, the model confirms that the atypical intervention succeeded in increasing expense predictions ($b = .21$, SE = .10, 95% CI = [.02, .40]; $t(591) = 2.16$, $p = .032$), and that higher predictions are associated with higher intended savings ($b = 25.30$, SE = 10.89, 95% CI = [3.92, 46.69]; $t(590) = 2.32$, $p = .021$).
